# Interfacial Covalent Bonds Regulated Electron‐Deficient 2D Black Phosphorus for Electrocatalytic Oxygen Reactions

**DOI:** 10.1002/adma.202008752

**Published:** 2021-05-03

**Authors:** Xia Wang, Ramya Kormath Madam Raghupathy, Christine Joy Querebillo, Zhongquan Liao, Dongqi Li, Kui Lin, Martin Hantusch, Zdeněk Sofer, Baohua Li, Ehrenfried Zschech, Inez M. Weidinger, Thomas D. Kühne, Hossein Mirhosseini, Minghao Yu, Xinliang Feng

**Affiliations:** ^1^ Center for Advancing Electronics Dresden (cfaed) & Faculty of Chemistry and Food Chemistry Technische Universität Dresden Mommsenstrasse 4 Dresden 01062 Germany; ^2^ Dynamics of Condensed Matter and Center for Sustainable Systems Design Chair of Theoretical Chemistry University of Paderborn Warburger Str. 100 Paderborn 33098 Germany; ^3^ Institute for Complex MateSrials Leibniz‐Institute for Solid State and Materials Research (IFW) Dresden 01069 Germany; ^4^ Fraunhofer Institute for Ceramic Technologies and Systems (IKTS) Maria‐Reiche‐Strasse 2 Dresden 01109 Germany; ^5^ Shenzhen Key Laboratory of Power Battery Safety and Shenzhen Geim Graphene Center Tsinghua Shenzhen International Graduate School Tsinghua University Shenzhen 518055 China; ^6^ Department of Inorganic Chemistry University of Chemistry and Technology Prague Technická 5 Prague 6 16628 Czech Republic

**Keywords:** 2D materials, bifunctional oxygen electrocatalysts, black phosphorus, oxygen evolution reaction, zinc–air batteries

## Abstract

Developing resource‐abundant and sustainable metal‐free bifunctional oxygen electrocatalysts is essential for the practical application of zinc–air batteries (ZABs). 2D black phosphorus (BP) with fully exposed atoms and active lone pair electrons can be promising for oxygen electrocatalysts, which, however, suffers from low catalytic activity and poor electrochemical stability. Herein, guided by density functional theory (DFT) calculations, an efficient metal‐free electrocatalyst is demonstrated via covalently bonding BP nanosheets with graphitic carbon nitride (denoted BP‐CN‐*c*). The polarized P—N covalent bonds in BP‐CN‐*c* can efficiently regulate the electron transfer from BP to graphitic carbon nitride and significantly promote the OOH* adsorption on phosphorus atoms. Impressively, the oxygen evolution reaction performance of BP‐CN‐*c* (overpotential of 350 mV at 10 mA cm^−2^, 90% retention after 10 h operation) represents the state‐of‐the‐art among the reported BP‐based metal‐free catalysts. Additionally, BP‐CN‐*c* exhibits a small half‐wave overpotential of 390 mV for oxygen reduction reaction, representing the first bifunctional BP‐based metal‐free oxygen catalyst. Moreover, ZABs are assembled incorporating BP‐CN‐*c* cathodes, delivering a substantially higher peak power density (168.3 mW cm^−2^) than the Pt/C+RuO_2_‐based ZABs (101.3 mW cm^−2^). The acquired insights into interfacial covalent bonds pave the way for the rational design of new and affordable metal‐free catalysts.

## Graphical abstract

A black‐phosphorus‐based metal‐free bifunctional oxygen electrocatalyst is developed by covalently bonding black phosphorus with graphitic carbon nitride. The interfacial P—N covalent bonds in the hybrid catalyst are highlighted to efficiently regulate the electron transfer from black phosphorus to graphitic carbon nitride and significantly promote the OOH* adsorption on phosphorus atoms.

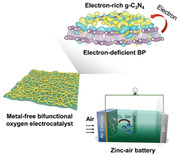

## 1 Introduction

Rechargeable zinc–air batteries (ZABs) have been recognized as one of the most promising energy technologies owing to their unique half‐closed configuration, large specific capacity (≈820 mAh g^−1^ based on Zn metal), high energy density (1086 Wh kg^−1^ based on Zn metal), and good environmental friendliness.^[^ ^1^, ^2^ ^]^ As the key component of ZABs, bifunctional electrocatalysts for oxygen evolution reaction (OER) and oxygen reduction reaction (ORR) are of importance to determine the charge/discharge kinetics and energy efficiency of ZABs.^[^ ^3^ ^]^ Currently, noble metal‐based (e.g., Pt, Ru, and Ir) electrocatalysts are the most prevalent category for oxygen electrocatalysis. However, their wide applications seriously suffer from the crustal scarcity and high cost.^[^ ^1^, ^4^ ^]^ In this regard, metal‐free catalysts are highly attractive because only low‐cost and resource‐abundant elements are utilized. Recently, extensive efforts have been devoted to developing carbon‐based metal‐free catalysts because the electronic structure of carbon materials can be elaborately regulated by heteroatom doping. Various heteroatom‐doped carbon materials have shown remarkable ORR activity at a level comparable to Pt/C (e.g., N‐doped graphene,^[^ ^5^ ^]^ B‐doped carbon nanotubes,^[^ ^6^ ^]^ P‐doped graphite,^[^ ^7^ ^]^ and S‐doped graphene^[^ ^8^ ^]^). Nevertheless, the employment of carbon‐based metal‐free catalysts in ZABs has been restricted by their unsatisfying OER performance, including the much higher overpotential than the benchmark catalysts (i.e., Ir/C and RuO_2_)^[^ ^9^ ^]^ and the poor catalytic stability due to the carbon corrosion at high anodic potentials.^[^ ^10^ ^]^ Therefore, developing metal‐free bifunctional catalysts based on other resource‐abundant elements is critically important to construct cost‐efficient and high‐performance ZABs.

Recently, 2D black phosphorus (BP) has emerged as a fascinating multifunctional material owing to its appealing features, such as the puckered‐honeycomb configuration, large surface‐to‐volume ratio, tunable bandgap (0.3–2.2 eV), and high charge carrier mobility (≈1000 cm^2^ V^−1^ s^−1^).^[^ ^11^, ^12^ ^]^ Particularly, the active lone pair electrons of P atoms offer favorable chemisorption sites for the O‐containing species (e.g., OH*, O*, and OOH*), empowering BP as a potential catalyst for OER.^[^ ^13^ ^]^ In an ideal case, all P atoms of single‐layer BP can be exposed to the surface and serve as active catalytic sites.^[^ ^14^, ^15^ ^]^ However, the OER activity of the early reported 2D BP‐based metal‐free catalysts is still far from satisfactory (overpotential of >550 mV at 10 mA cm^−2^),^[^ ^14^ ^] [^ ^16^ ^]^ due to the low adsorption/dissociation kinetics of O‐intermediates on BP. Moreover, the active lone pair electrons make BP prone to be oxidized, especially when the OER process provides a strong oxidative environment with a high anodic potential.^[^ ^17^, ^18^ ^]^ Thus, exploiting effective strategies to regulate the electronic structure of BP will be highly desirable for improving the OER activity and stability. Besides, the development of BP‐based metal‐free bifunctional catalysts toward OER and ORR remains a blank to be filled.

Herein, we report the first BP‐based metal‐free bifunctional oxygen electrocatalyst (denoted BP‐CN‐*c*) by covalently bonding BP with graphitic carbon nitride (g‐C_3_N_4_). Polarized P—N covalent bonds are unveiled to regulate the electron redistribution among the heterointerfaces, inducing electron transfer from P atoms to g‐C_3_N_4_. The manipulated electron‐deficient feature empowers BP with considerably enhanced OOH* chemisorption capability and chemical stability. Remarkably, the BP‐CN‐*c* catalyst presents a high OER activity with a small overpotential of 350 mV at 10 mA cm^−2^, which outperforms all the previously reported BP‐based metal‐free catalysts. Moreover, BP‐CN‐*c* presents excellent OER stability with only 10% current loss after 10 h continuous operation. Besides, the BP‐CN‐*c* catalyst achieves a superb ORR performance in 0.1 m KOH solution, depicting a halfwave overpotential (390 mV), well comparable to the state‐of‐the‐art Pt/C. As a result, ZABs are constructed to demonstrate the feasibility of BP‐CN‐*c* in practical applications. A peak power density of 168.3 mW cm^−2^ is reached by ZABs based on the BP‐CN‐*c* cathodes, which significantly outweighs those based on the commercial Pt/C+RuO_2_ catalyst (101.3 mW cm^−2^).

## 2 Results and Discussion

**Figure 1:**
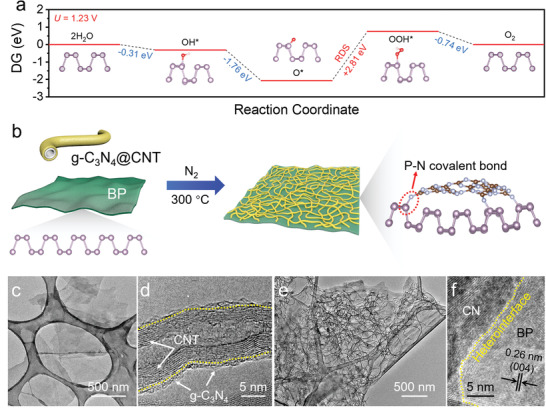
a) The calculated Gibbs free energy diagrams of the OER process on BP at *U* = 1.23 V. The purple, light blue, and brown balls represent P, N, and C atoms, respectively. b) Schematic illustration of BP‐CN‐*c*. c–f) TEM images of BP (c), CN (d), and BP‐CN‐*c* (e,f).

Moderate adsorption (neither too strong nor too weak) of adsorbates on catalytic sites is generally considered as the key prerequisite for an outstanding electrocatalyst. To understand the inferior OER performance of pristine BP, we first performed density functional theory (DFT) calculations to elucidate the O‐intermediates binding energies (Δ*G*) on BP during the OER process (**Figure** **1**a). Except for the step from O* to OOH*, all the other steps of the OER process are spontaneous at the potential (*U*) of 1.23 V. The formation of OOH* with a large energy barrier of 2.81 eV is thereby the potential rate‐determining step (RDS) of the OER process. Moreover, calculations are performed for oxygen‐terminated BP (denoted BP‐O), as BP‐O is recognized to be the stabilized configuration of BP when exposed to air.^[^ ^19^ ^]^ Similarly, the formation of OOH* is confirmed as the RDS for the OER process on BP‐O, showing a large energy barrier of 3.25 eV at *U* = 1.23 V (Figure S1, Supporting Information). These results suggest that modulating the local electronic structure of BP to promote the OOH* adsorption is the key to boost its OER performance.

Inspired by the heteroatom doping strategy for carbon‐based electrocatalysts, we hypothesize that covalently bonding BP surface to other heteroatoms would alter the electron redistribution and thus tailor the adsorption energies of O‐intermediates on BP. In this scenario, graphitic carbon nitride (g‐C_3_N_4_) was selected to react with BP sheets because the rich pyridinic‐N and graphitic‐N sites allow the formation of polarized P—N covalent bonds at the BP/g‐C_3_N_4_ interfaces. Figure 1b illustrates the structural configuration of our developed BP‐CN‐*c* catalyst. Few‐layer BP nanosheets with lateral sizes of 1–10 µm (Figure 1c; Figure S2, Supporting Information) and thicknesses of 1.3–9.5 nm (mean thickness of 3.7 ± 1.3 nm) were first prepared via an electrochemical delamination method reported in our previous work.^[^ ^12^ ^]^ Subsequently, g‐C_3_N_4_ nanosheets were grown on multiwalled carbon nanotubes (CNTs) to enable a good electrical conductivity (the hybrid is denoted CN). To promote the formation of P—N covalent bonds at the BP/g‐C_3_N_4_ interfaces, BP nanosheets and CN were mixed thoroughly and then subjected to annealing at 300 °C under N_2_ atmosphere. The annealing temperature of 300°C was selected to ensure the formation of rich P—N covalent bonds (Figure S3, Supporting Information), while avoiding the thermal decomposition of g‐C_3_N_4_ and BP (Figure S4, Supporting Information).

Transmission electron microscopy (TEM) images (Figure 1d; Figure S5, Supporting Information) verify the uniform coating of ultrathin g‐C_3_N_4_ nanosheets on the surface of CNTs. Fourier transform infrared (FTIR) spectroscopy (Figure S6, Supporting Information), X‐ray diffraction (Figure S7, Supporting Information), and SEM images (Figure S8, Supporting Information) also manifest the successful growth of g‐C_3_N_4_ nanosheets on the surface of CNTs. Such a 1D g‐C_3_N_4_/CNT hybrid can serve as the spacer to prevent the re‐stacking when coupled with BP nanosheets, thus enabling the maximum formation of BP/g‐C_3_N_4_ heterointerfaces. As revealed by TEM images (Figure 1e,f) and energy dispersive X‐ray (EDX) spectroscopy elemental mapping (Figure S9, Supporting Information), CN is homogenously and densely distributed over BP nanosheets, exposing abundant heterointerfaces. Moreover, BP‐CN‐*c* exhibits a specific surface area of 119 m^2^ g^–1^ (Figure S10, Supporting Information).

**Figure 2:**
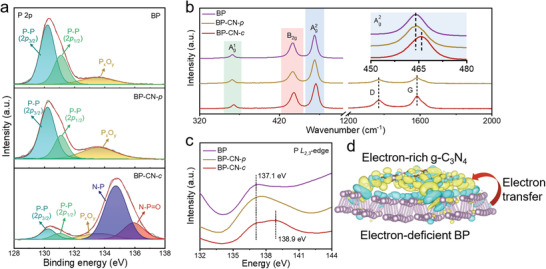
a) P 2p XPS, b) Raman, and c) P *L*_2,3_‐edge XANES spectra of BP, BP‐CN‐*p*, and BP‐CN‐*c*. d) The optimized atomic configuration and electron‐density difference isosurfaces of the P—N bonded BP/g‐C_3_N_4_ hybrid. The purple, brown, and light blue balls represent P, C, and N atoms, respectively. The yellow and cyan regions correspond to electron accumulation and depletion, respectively (isosurface of 0.00185 e Å^−3^).

To highlight the vital role of P—N covalent bonds in regulating the electron redistribution at the BP/g‐C_3_N_4_ interface, physically mixed BP and CN (denoted BP‐CN‐*p*) was prepared as a control sample. X‐ray photoelectron spectroscopy (XPS) provides direct evidence for the formation of P—N bonds in BP‐CN‐*c*. In addition to P‐P 2p_3/2_ peak (130.3 eV), P‐P 2p_1/2_ peak (131.1 eV) peak, and a broad oxidation peak (133.6 eV) of BP, N—P (134.7 eV), and N—P=O (135.9 eV) peaks are detected in the P 2p XPS of BP‐CN‐*c* (**Figure** **2**a).^[^ ^20^ ^]^ Of note, these two peaks were not observed for BP‐CN‐*p*. Additionally, N 1s XPS (Figure S11, Supporting Information) and FTIR spectra (Figure S6, Supporting Information) also confirm the formation of P—N bonds in BP‐CN‐*c*, rather than in BP‐CN‐*p*. The strong interfacial interaction in BP‐CN‐*c* via P—N bonds induces the negative shift of the N‐C peak in C 1s XPS spectra by 0.3 eV, which is a sign of electron transfer from BP to g‐C_3_N_4_ (Figure S12, Supporting Information).^[^ ^18^, ^21^ ^]^ Figure 2b compares the Raman spectra of BP, BP‐CN‐*p*, and BP‐CN‐*c*, which all display three characteristic peaks located at 362 (A^1^_g_ out‐of‐plane vibrational mode), 438 (B_2g_ armchair vibrational mode), and 464–466 cm^−1^ (and A^2^_g_ zig‐zag vibrational mode).^[^ ^22^ ^]^ In comparison to BP and BP‐CN‐*p* (464 cm^−1^), BP‐CN‐*c* (466 cm^−1^) shows an apparent positive shift in the A^2^_g_ peak, implying that the interfacial interaction in BP‐CN‐*c* introduces strong strain into the BP plane.^[^ ^23^ ^]^ The P *L*_2,3_‐edge X‐ray absorption near edge structure spectra (XANES) spectra further provide insights into the local electronic structures of P atoms in different samples (Figure 2c). Peaks ranging from 135.0 to 141.0 eV refer to transitions from 2p electrons of P into the first unoccupied 3s antibonding state. Among, a peak centered at 137.1 eV can be observed for BP, BP‐CN‐*p*, and BP‐CN‐*c*, corresponding to P—O species formed by the slight oxidation of BP.^[^ ^24^ ^]^ Unlike BP and BP‐CN‐*p*, BP‐CN‐*c* displays a distinguishable peak at 138.9 eV, which supports the formation of P—N covalent bonds.^[^ ^25^ ^]^

Besides, DFT simulations were performed to investigate the atomic/electronic structure of the P—N bonded BP/g‐C_3_N_4_ hybrid (Figures S13 and S14, Supporting Information). Figure 2d displays the most stable atomic configuration with computed charge‐density difference isosurfaces. As expected, the optimized BP/g‐C_3_N_4_ structure is featured with a slightly deformed BP plane and noticeable electron depletion in P atoms, which agrees well with the experimental analysis. Thereby, we can conclude that the polarized P—N covalent bonds can significantly influence the electron redistribution at BP/g‐C_3_N_4_ interfaces, which produces electron‐deficient BP planes. In addition, it was recognized that the lone pair electrons of sp^3^‐hybridized P in BP were susceptible to bond with chemisorbed oxygen atoms and be oxidized.^[^ ^16^ ^]^ By forming P—N covalent bonds, the active lone pair electrons of P in BP‐CN‐*c* are substantially occupied, thus prominently promting the stability of BP‐CN‐*c* (Figure S15, Supporting Information).

**Figure 3:**
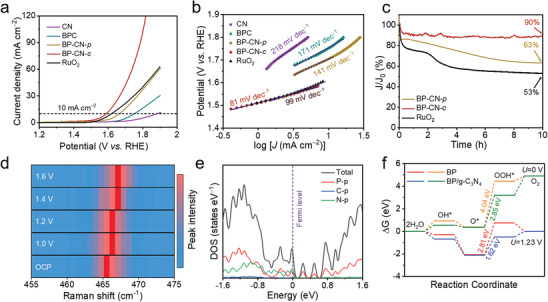
a) OER polarization curves and b) Tafel plots of CN, BPC, BP‐CN‐*p*, BP‐CN‐*c*, and RuO_2_. c) Chronoamperometric curves of BP‐CN‐*p*, BP‐CN‐*c*, and RuO_2_ at 1.58 V versus RHE. d) In situ Raman spectro‐electrochemistry of BP‐CN*‐c* A^2^_g_ band at various potentials (vs RHE). e) The calculated DOS of the P—N bonded BP/g‐C_3_N_4_ hybrid. f) The free energy diagrams of the OER process on BP and the BP/g‐C_3_N_4_ hybrid at *U* = 0 and 1.23 V.

The modified electronic structure of P atoms in BP‐CN‐*c* motivated us to assess the OER catalytic performance of BP‐CN‐*c*. It should be noted that, to obtain the optimal catalytic performance, the urea‐to‐CNTs ratio used for synthesizing CN (Table S1 and Figure S16, Supporting Information) and the BP‐to‐CN ratio used for synthesizing BP‐CN‐*c* (Figure S17, Supporting Information) were rationally optimized. The significiant role of CNTs in BP‐CN‐*c* is also highlighted by the poor catalytic performance of the directly hybrided BP and g‐C_3_N_4_ (Figure S18, Supporting Information). **Figure** **3**a presents the linear sweep voltammetry (LSV) polarization curves of CN, the mixture of BP and CNTs (denoted as BPC), BP‐CN‐*p*, BP‐CN‐*c*, and commercial RuO_2_ in 1 m N_2_‐saturated KOH solution. Clearly, the as‐prepared BP‐CN‐*c* catalyst outperforms all other metal‐free counterparts and the benchmark RuO_2_ catalyst. A current density of 10 mA cm^−2^ is achieved for the BP‐CN‐*c* catalyst with an overpotential of 350 mV, while the overpotentials for CN, BPC, BP‐CN‐*p*, and RuO_2_ are 650, 520, 430, and 385 mV, respectively (Figure 3a). The smallest Tafel slope (81 mV dec^−1^, Figure 3b) and the highest electrochemically active surface area (16.58 mF cm^–2^, Figures S19 and S20, Supporting Information) of BP‐CN‐*c* also reflect its superior OER kinetics and activity to other counterparts. Excellent reproducibility was demonstrated for the OER performance of BP‐CN‐*c* (Figure S21, Supporting Information). Significantly, the OER activity of BP‐CN‐*c* represents the best among the reported BP‐based OER catalysts (Table S2, Supporting Information).

In addition, the N—P bond in BP‐CN‐*c* greatly improve the resistance of BP against structure degradation during the long‐term OER operation. In a chronoamperometric test at 1.58 V for 10 h, BP‐CN‐*c* exhibits high current retention of ≈90%, which substantially outperforms BP‐CN‐*p* (63%) and RuO_2_ (53%) (Figure 3c). Moreover, BP‐CN‐*c* still retains high current retention of 86.3% after the 20 h chronoamperometric test (Figure S22, Supporting Information). We further examined the stability through a standard accelerated durability test with a scan cycling potential window between 1.3 and 1.7 V versus reversible hydrogen electrode (RHE). After 1000 cycles, the OER overpotential of BP‐CN‐*c* at 10 mA cm^−2^ exhibited a negligible increase of less than 1 mV, which is much better than that of BP‐CN‐*p* with an increase of 40 mV (Figure S23, Supporting Information).

In situ Raman spectro‐electrochemistry of BP‐CN‐*c* during the OER process elucidates that P atoms are active catalytic sites for the OER (Figure 3d; Figure S24, Supporting Information). When the imposed potential increases from the open circuit potential (OCP, 0.875 V vs RHE) to 1.0 V versus RHE, the slight positive shift of the A_g_^2^ peak can be explained by the compressive strain in BP planes induced by the adsorption of OH^−^ on P atoms.^[^ ^26^ ^]^ A further positive shift can be observed for the A_g_^2^ peak at a potential exceeding the thermodynamic OER potential (1.4 V vs RHE), which can be assigned to the conversion of the adsorbed O‐intermediates.^[^ ^23^ ^]^ DFT calculations further provide insights into how P—N bonds promote the OER performance of the BP‐CN‐*c* catalyst. Figure 3e displays the calculated density of states (DOS) for the P—N bonded BP/g‐C_3_N_4_. Impressively, the Fermi level (EF) of the BP/g‐C_3_N_4_ heterostructure lies inside the bands, indicating the metallic feature of BP/g‐C_3_N_4_. The BP/g‐C_3_N_4_ interface produces interface states that are composed of N 2p and P 3p states. This is due to the covalent bonding between N and P in the BP/g‐C_3_N_4_ heterostructure. The appearance of interface states in the bandgap results in the metallic feature, which can significantly improve the catalytic performance of the BP/g‐C_3_N_4_ heterostructure compared with semiconducting BP and g‐C_3_N_4_ (Figure S25, Supporting Information). The free energy diagrams of the OER process on BP and BP/g‐C_3_N_4_ hybrid at *U* = 0 and 1.23 V are summarized in Figure 3f. As discussed earlier, the formation of OOH* is regarded as the RDS for BP. In comparison with BP (4.04 eV at *U* = 0 V and 2.81 eV at *U* = 1.23 V), the BP/g‐C_3_N_4_ hybrid presents a much lower energy barrier for the OOH* formation (2.85 eV at *U* = 0 V and 1.62 eV at *U* = 1.23 V), significantly promoting the conversion from O* to OOH*. Similar calculations were also performed for BP‐O and the BP‐O/g‐C_3_N_4_ hybrid, which offer the same conclusion for the role of P—N bonds (Figures S26 and S27, Supporting Information).

**Figure 4:**
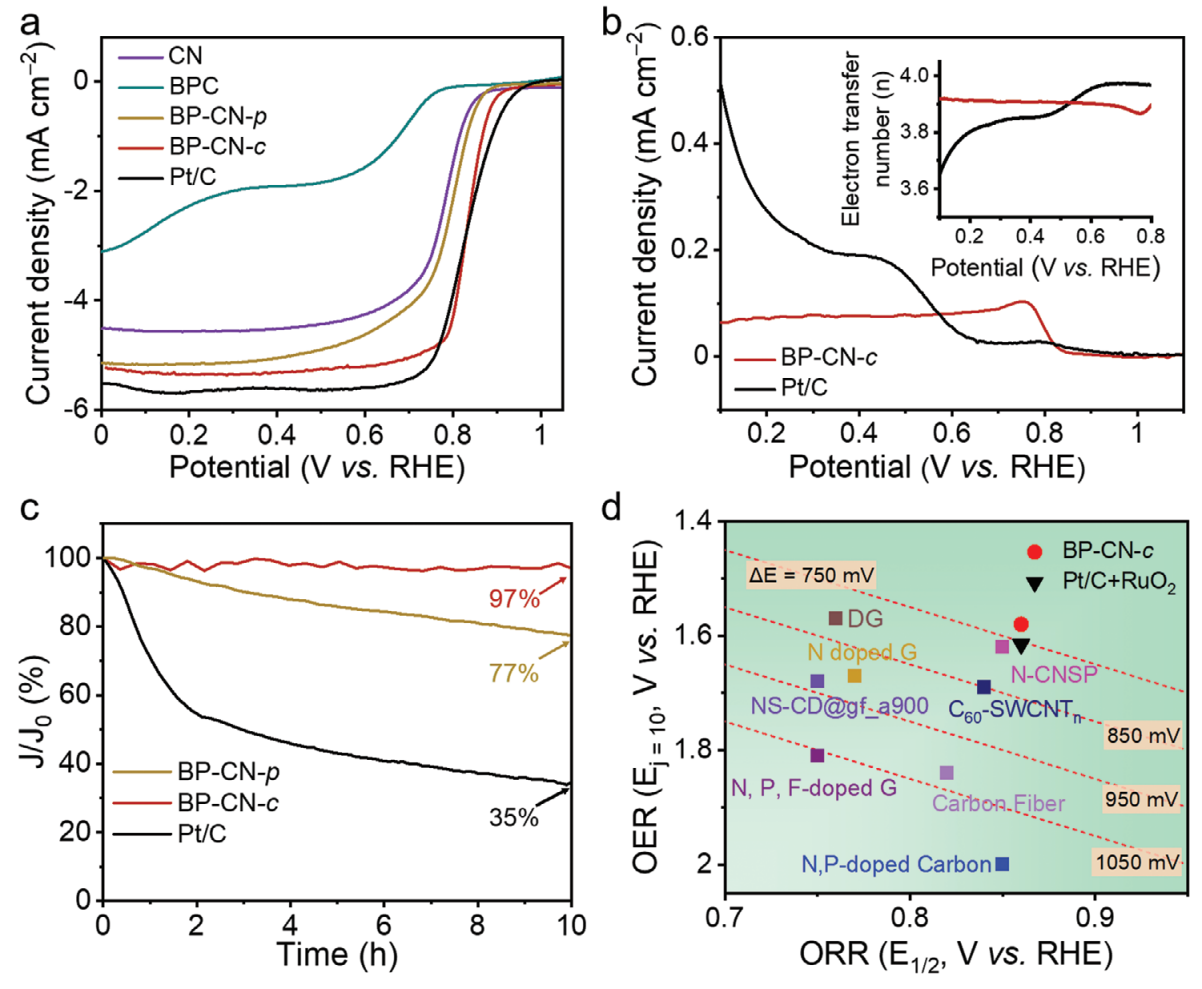
a) ORR polarization curves of CN, BPC, BP‐CN‐*p*, BP‐CN‐*c*, and Pt/C at 5 mV s^−1^ with a rotating speed of 1600 rpm. b) Ring current densities and the calculated electron transfer numbers (inset) for BP‐CN‐*c*, and Pt/C during ORR process. c) Chronoamperometric curves of BP‐CN*‐p*, BP‐CN*‐c*, and Pt/C at 0.7 V vs RHE. d) OER and ORR activities of BP‐CN*‐c* in 1 m KOH compared with Pt/C+RuO_2_ and representative metal‐free catalysts.^[^ ^27^, ^28^, ^29^, ^30^, ^31^, ^32^, ^33^ ^]^ The dotted lines refer to Δ*E* at the same values.

Furthermore, the BP‐CN‐*c* catalyst shows a comparable ORR catalytic activity to the commercial Pt/C in an O_2_‐saturated 0.1 m KOH solution. The cyclic voltammetry curve (Figure S28, Supporting Information) of BP‐CN‐*c* shows a distinct cathodic peak centered at ≈0.80 V in the O_2_‐saturated 0.1 m KOH electrolyte, indicating the pronounced electrocatalytic activity for ORR. LSV curves at 1600 rpm indicate that the BP‐CN‐*c* catalyst achieves a half‐wave potential of 0.84 V versus RHE and a diffusion‐limiting current density of 5.34 mA cm^−2^ (**Figure** **4**a). This catalytic performance is well comparable to that of Pt/C (0.85 V vs RHE, 5.60 mA cm^−2^), and considerably better than those of BPC (0.68 V vs RHE, 1.92 mA cm^−2^), CN (0.81 V vs RHE, 5.15 mA cm^−2^), and BP‐CN‐*p* (0.80 V vs RHE, 4.55 mA cm^−2^). In addition, the good ORR reproducibility of the BP‐CN‐*c* catalyst is supported by the LSV curves collected from four RDEs with BP‐CN‐*c* prepared from the same batch and BP‐CN‐*c* prepared from different batches (Figure S29, Supporting Information). Meanwhile, the electron transfer number of ORR on BP‐CN‐*c* was measured by rotating ring‐disk electrode measurements (Figure S30a,b, Supporting Information). A close‐to‐4‐electron (3.87–3.92 electrons) pathway could be verified at 0.8–0.1 V versus RHE, demonstrating that O_2_ was almost completely reduced to H_2_O (Figure 4b). RDE polarization curves at a variety of rotating speeds were also collected for BP‐CN‐*c*, which identify the first‐order reaction kinetics of BP‐CN‐*c* concerning the concentration of dissolved oxygen (Figure S30c, Supporting Information). Moreover, BP‐CN‐*c* exhibited a remarkable ORR durability with a negligible current loss (3%) at 0.7 V versus RHE for 10 h ORR operation (Figure 4c). This result stands in contrast to the poor durability of BP‐CN‐*p* and Pt/C catalysts with large current decay of 23% and 55%, respectively. The superior ORR activity of the BP‐CN‐*c* catalyst can be attributed to the accelerated electron transport of the P—N bonded BP/g‐C_3_N_4_ hybrid and the reduced energy barriers of ORR steps occurring on carbon atoms of g‐C_3_N_4_ (Figure S31, Supporting Information).

Next, the bifunctional catalytic performance of BP‐CN‐*c* was assessed by calculating the potential gap (Δ*E*) between the OER potential at 10 mA cm^−2^ (*E* *_j_*_= 10_) and ORR half‐wave potential (*E*_1/2_) in 1 m KOH (Figure S32, Supporting Information). For comparison, Δ*E* of the benchmarked Pt/C+RuO_2_ catalyst and recently reported metal‐free catalysts are also summarized in Figure 4d. Impressively, the BP‐CN‐*c* catalyst achieves a small Δ*E* of 720 mV, which is not only better than Pt/C+RuO_2_ (755 mV), but also outperforms most of the previously reported metal‐free catalysts, such as N,P‐doped carbon (1150 mV),^[^ ^33^ ^]^ carbon fiber (1020 mV),^[^ ^32^ ^]^ N,P,F‐doped G (1060 mV),^[^ ^30^ ^]^ C_60_‐SWCNTn (850 mV),^[^ ^29^ ^]^ NS‐CD@gf_a900 (930 mV),^[^ ^28^ ^]^ N‐doped G (900 mV),^[^ ^31^ ^]^ DG (810 mV),^[^ ^30^ ^]^ and N‐CNSP (765 mV).^[^ ^27^ ^]^ All these results suggest that BP‐CN‐*c* is a promising metal‐free bifunctional catalyst.

**Figure 5:**
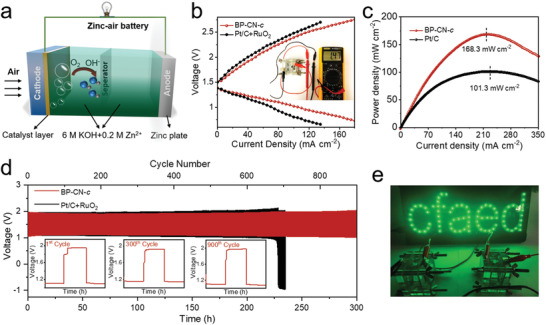
a) Schematic illustration of rechargeable ZABs based on the BP‐CN‐*c* catalyst. b) Discharge/charge polarization curves, c) power density curves, and d) discharge/charge cycling curves at 10 mA cm^−2^ of BP‐CN‐*c*‐ and Pt/C+RuO_2_‐based ZABs. e) Digital photo of a green LED board powered by two BP‐CN‐*c*‐based ZABs connected in series.

Rechargeable ZABs were subsequently assembled by employing BP‐CN*‐c* as the catalyst for cathodes (**Figure** **5**a). For the sake of comparison, reference ZABs were assembled by using Pt/C+RuO_2_ catalyst (Pt/Ru 1:1 mass ratio) for cathodes. The open‐circuit voltage of BP‐CN*‐c*‐based ZABs achieved 1.47 V (inset of Figure 5b; Figure S33, Supporting Information). Due to the excellent bifunctional activity of BP‐CN*‐c*, BP‐CN*‐c*‐based ZABs exhibited apparently narrower charge/discharge voltage gaps than Pt/C+RuO_2_‐based ZABs in a broad current density range (1–180 mA cm^−2^) (Figure 5b). Much higher discharging plateaus were also detected for BP‐CN*‐c*‐based ZABs than Pt/C+RuO_2_‐based ZABs at the same current densities (Figure S34, Supporting Information). Power density curves were further collected for BP‐CN*‐c*‐based ZABs and Pt/C+RuO_2_‐based ZABs by the LSV test with a scan rate of 5 mV s^−1^ from 1.55 to 0.2 V. The peak power density of BP‐CN*‐c*‐based ZABs reached 168.3 mW cm^−2^, which is much higher than that of Pt/C+RuO_2_‐based ZABs (101.3 mW cm^−2^) (Figure 5c). Four additional BP‐CN‐*c*‐based ZABs were fabricated with catalysts prepared from different batches, showing repeatable battery performance (Figure S35, Supporting Information). In addition, the power density curves derived from Figure 5b also indicate the superior performance of BP‐CN‐*c*‐based ZABs compared with Pt/C+RuO_2_‐based ZABs (Figure S36, Supporting Information). Remarkably, the power performance of BP‐CN*‐c*‐based ZABs surpasses most of recently reported ZABs based on the metal‐free bifunctional catalysts (Table S3, Supporting Information). Besides, the specific capacity and energy density of BP‐CN*‐c*‐based ZABs reached 793.9 mAh g^−1^ and 952.7 Wh kg^−1^ (based on the mass of Zn metal) at a discharge current density of 5 mA cm^−2^, which also outclasses those of Pt/C+RuO_2_‐based ZABs (751.3 mAh g^−1^ and 826.4 Wh kg^−1^) (Figure S37, Supporting Information).

The cycling stability of the fabricated ZABs was further examined at a current density of 10 mA cm^−2^ with 20 min per cycle (10 min for discharge, 10 min for charge). In the first cycle, the discharge and charge voltages of BP‐CN*‐c*‐based ZABs were 1.10 and 1.95 V, respectively (Figure 5d). After 900 cycles (operation for 300 h), the devices still featured a stable discharge voltage of 1.09 V and a charge voltage of 1.98 V. In sharp contrast, battery failure was observed for Pt/C+RuO_2_‐based ZABs after 700 cycles. Finally, two BP‐CN*‐c*‐based ZABs were connected in series, by which a green light emitting diode (LED) with an operating voltage of 2.5 V could be successfully lighted up (Figure 5e). This result demonstrates the feasibility of BP‐CN*‐c*‐based ZABs for practical use.

## 3 Conclusion

We have developed the first 2D BP‐based metal‐free bifunctional oxygen catalyst. Experimental studies accompanied with DFT calculations corroborated the vital role of interfacial P—N bonds in regulating the electron redistribution between BP/g‐C_3_N_4_ interfaces and thus the chemisorption properties of O‐intermediates. The as‐developed BP‐CN‐*c* catalyst achieved the best OER catalytic performance among BP‐based metal‐free catalysts and a comparable ORR catalytic activity to the commercial Pt/C catalyst. Additionally, the application of BP‐CN‐*c* for ZAB cathodes was also demonstrated, which considerably surpasses the benchmark Pt/C+RuO_2_ catalyst. This work emphasizes that interfacial covalent bond engineering can be a powerful strategy for devising bifunctional metal‐free hybrid catalysts required by diverse energy storage and conversion applications.

## Conflict of Interest

The authors declare no conflict of interest.

## Supporting information

Supporting Information

## Data Availability

Research data are not shared.

## References

[1] Y. P. Zhu , C. Guo , Y. Zheng , S.‐Z. Qiao , Acc. Chem. Res. 2017, 50, 915. 28205437 10.1021/acs.accounts.6b00635

[2] a) M. Yu , R. Dong , X. Feng , J. Am. Chem. Soc. 2020, 142, 12903; 32628838 10.1021/jacs.0c05130

[3] a) L. Zhu , D. Zheng , Z. Wang , X. Zheng , P. Fang , J. Zhu , M. Yu , Y. Tong , X. Lu , Adv. Mater. 2018, 30, 1805268; 10.1002/adma.201805268 30259586

[4] a) D. Zhao , Z. Zhuang , X. Cao , C. Zhang , Q. Peng , C. Chen , Y. Li , Chem. Soc. Rev. 2020, 49, 2215; 32133461 10.1039/c9cs00869a

[5] L. Qu , Y. Liu , J.‐B. Baek , L. Dai , ACS Nano 2010, 4, 1321. 20155972 10.1021/nn901850u

[6] L. Yang , S. Jiang , Y. Zhao , L. Zhu , S. Chen , X. Wang , Q. Wu , J. Ma , Y. Ma , Z. Hu , Angew. Chem., Int. Ed. 2011, 50, 7132. 10.1002/anie.201101287 21688363

[7] Z. W. Liu , F. Peng , H. J. Wang , H. Yu , W. X. Zheng , J. Yang , Angew. Chem., Int. Ed. 2011, 50, 3257. 10.1002/anie.201006768 21381161

[8] I. Y. Jeon , S. Zhang , L. Zhang , H. J. Choi , J. M. Seo , Z. Xia , L. Dai , J. B. Baek , Adv. Mater. 2013, 25, 6138. 24038522 10.1002/adma.201302753

[9] X. Liu , L. Dai , Nat. Rev. Mater. 2016, 1, 16064.

[10] a) N. Giordano , P. Antonucci , E. Passalacqua , L. Pino , A. Arico , K. Kinoshita , Electrochim. Acta 1991, 36, 1931;

[11] a) J. Kang , S. A. Wells , J. D. Wood , J.‐H. Lee , X. Liu , C. R. Ryder , J. Zhu , J. R. Guest , C. A. Husko , M. C. Hersam , Proc. Natl. Acad. Sci. USA 2016, 113, 11688; 27092006 10.1073/pnas.1602215113 PMC5081619

[12] S. Yang , K. Zhang , A. G. Ricciardulli , P. Zhang , Z. Liao , M. R. Lohe , E. Zschech , P. W. M. Blom , W. Pisula , K. Mullen , X. Feng , Angew. Chem., Int. Ed. 2018, 57, 4677. 10.1002/anie.201801265 29474753

[13] a) Q. Jiang , L. Xu , N. Chen , H. Zhang , L. Dai , S. Wang , Angew. Chem., Int. Ed. 2016, 55, 13849; 10.1002/anie.201607393 27682470

[14] X. Ren , J. Zhou , X. Qi , Y. Liu , Z. Huang , Z. Li , Y. Ge , S. C. Dhanabalan , J. S. Ponraj , S. Wang , Adv. Energy Mater. 2017, 7, 1700396.

[15] L. Zhang , L. X. Ding , G. F. Chen , X. Yang , H. Wang , Angew. Chem., Int. Ed. 2019, 58, 2612. 10.1002/anie.201813174 30560583

[16] T. Yin , L. Long , X. Tang , M. Qiu , W. Liang , R. Cao , Q. Zhang , D. Wang , H. Zhang , Adv. Sci. 2020, 7, 2001431. 10.1002/advs.202001431 PMC7539224 33042754

[17] a) Z. Hu , Q. Li , B. Lei , Q. Zhou , D. Xiang , Z. Lyu , F. Hu , J. Wang , Y. Ren , R. Guo , Angew. Chem., Int. Ed. 2017, 56, 9131; 10.1002/anie.201705012 28627084

[18] X. Zhu , T. Zhang , D. Jiang , H. Duan , Z. Sun , M. Zhang , H. Jin , R. Guan , Y. Liu , M. Chen , H. Ji , P. Du , W. Yan , S. Wei , Y. Lu , S. Yang , Nat. Commun. 2018, 9, 4177. 30301894 10.1038/s41467-018-06437-1 PMC6177470

[19] C. R. Ryder , J. D. Wood , S. A. Wells , Y. Yang , D. Jariwala , T. J. Marks , G. C. Schatz , M. C. Hersam , Nat. Chem. 2016, 8, 597. 27219705 10.1038/nchem.2505

[20] J. Holoubek , Y. Yin , M. Li , M. Yu , Y. S. Meng , P. Liu , Z. Chen , Angew. Chem., Int. Ed. 2019, 58, 18892. 10.1002/anie.201912167 31654444

[21] R. He , J. Hua , A. Zhang , C. Wang , J. Peng , W. Chen , J. Zeng , Nano Lett. 2017, 17, 4311. 28605201 10.1021/acs.nanolett.7b01334

[22] S. Sugai , I. Shirotani , Solid State Commun. 1985, 53, 753.

[23] P. Nakhanivej , X. Yu , S. K. Park , S. Kim , J.‐Y. Hong , H. J. Kim , W. Lee , J. Y. Hwang , J. E. Yang , C. Wolverton , Nat. Mater. 2019, 18, 156. 30531848 10.1038/s41563-018-0230-2

[24] Z. Zhang , P. Zhang , S. Yang , T. Zhang , M. Loffler , H. Shi , M. R. Lohe , X. Feng , Proc. Natl. Acad. Sci. USA 2020, 117, 13959. 32513735 10.1073/pnas.2003898117 PMC7321993

[25] a) G. Nicotra , A. Politano , A. Mio , I. Deretzis , J. Hu , Z. Mao , J. Wei , A. La Magna , C. Spinella , Phys. Status Solidi B 2016, 253, 2509;

[26] S. Sugai , T. Ueda , K. Murase , J. Phys. Soc. Jpn. 1981, 50, 3356.

[27] L. Zong , W. Wu , S. Liu , H. Yin , Y. Chen , C. Liu , K. Fan , X. Zhao , X. Chen , F. Wang , Energy Storage Mater. 2020, 27, 514.

[28] J. Shin , J. Guo , T. Zhao , Z. Guo , Small 2019, 15, 1900296. 10.1002/smll.201900296 30908886

[29] R. Gao , Q. Dai , F. Du , D. Yan , L. Dai , J. Am. Chem. Soc. 2019, 141, 11658. 31241328 10.1021/jacs.9b05006

[30] Y. Jia , L. Zhang , A. Du , G. Gao , J. Chen , X. Yan , C. L. Brown , X. Yao , Adv. Mater. 2016, 28, 9532. 27622869 10.1002/adma.201602912

[31] C. Tang , H. F. Wang , X. Chen , B. Q. Li , T. Z. Hou , B. Zhang , Q. Zhang , M. M. Titirici , F. Wei , Adv. Mater. 2016, 28, 6845. 27167616 10.1002/adma.201601406

[32] Q. Liu , Y. Wang , L. Dai , J. Yao , Adv. Mater. 2016, 28, 3000. 26914270 10.1002/adma.201506112

[33] J. Zhang , Z. Zhao , Z. Xia , L. Dai , Nat. Nanotechnol. 2015, 10, 444. 25849787 10.1038/nnano.2015.48

