# Supplementary material for: Interfacial Covalent Bonds Regulated Electron‐Deficient 2D Black Phosphorus for Electrocatalytic Oxygen Reactions
Source: Adv Mater. 2021 May 3;33(20):2008752. doi: 10.1002/adma.202008752 (PMC11469023; doi:10.1002/adma.202008752)
Supplement: Supplementary file 1 — Supporting Information [file ADMA-33-2008752-s001.pdf]

© 2021 Wiley-VCH GmbH

ADVANCED MATERIALS

Supporting Information

for *Adv. Mater.,* DOI: 10.1002/adma.202008752

Interfacial Covalent Bonds Regulated Electron-Deficient 2D Black Phosphorus for Electrocatalytic Oxygen Reactions

Xia Wang, Ramya Kormath Madam Raghupathy, Christine Joy Querebillo, Zhongquan Liao, Dongqi Li, Kui Lin, Martin Hantusch, Zdeněk Sofer, Baohua Li, Ehrenfried Zschech, Inez M. Weidinger, Thomas D. Kühne, Hossein Mirhosseini, Minghao Yu, and Xinliang Feng**

Supporting Information

Interfacial Covalent Bonds Regulated Electron-Deficient 2D Black Phosphorus for Electrocatalytic Oxygen Reactions

Xia Wang, Ramya Kormath Madam Raghupathy, Christine Joy Querebillo, Zhongquan Liao, Dongqi Li, Kui Lin, Martin Hantusch, Zdeněk Sofer, Baohua Li, Ehrenfried Zschech, Inez M. Weidinger, Thomas D. Kühne, Hossein Mirhosseini, Minghao Yu and Xinliang Feng**

Experimental section

Chemicals: Black phosphorus (BP) was purchased from smart-elements GmbH. Urea, carboxylic acid functionalized multiwall carbon nanotubes (CNTs), tetra-n-butyl-ammonium bisulfate (TBA·HSO_4), propylene carbonate (PC), and zinc acetate were purchased from Sigma Aldrich. Potassium hydroxide was purchased from Fisher Scientific. All the chemicals were used directly without any further purification.

Preparation of BP nanosheets: BP nanosheets were synthesized through an electrochemical exfoliation method reported by our group.[1] The electrochemical delamination of bulk BP crystal was conducted using a two-electrode system, in which a piece of platinum foil was used as the counter electrode and a bulk BP crystal was used as the working electrode. The electrolyte consisted of 0.1 M TBA·HSO_4 in anhydrous PC. The delamination process was conducted with a constant voltage of −8.5 V for 3.5 hours under the protection of inert Ar gas. After delamination, the exfoliated BP flakes were washed by anhydrous ethanol for 4 times, and then dispersed into deoxygenated anhydrous ethanol for further use.

Preparation of the CN samples: CN were prepared by annealing a mixture of urea and CNTs with different urea-to-CNTs ratios (CN8, CN16, CN24, and CN32 refer to the CN samples with urea-to-CNTs ratios of 8, 16, 24, and 32, respectively). For CN8, 50 mg of CNTs was dispersed in 100 mL water, and then 400 mg urea was added and stirred for 2 hours. Afterwards, the solvent was removed by rotary evaporation, and the collected solid mixture was thermal treated at 550 °C with a ramp rate of 2 °C min^{-1} for 4 hours. After cooling down to room temperature, CN8 was obtained by ground into powder using an agate mortar. CN16, CN24, and CN32 were prepared by a similar procedure except using different urea-to-CNTs ratios. In the manuscript, CN refers to the optimal sample, which is CN24.

Preparation of BP-CN-p and BP-CN-c: BP-CN-*p* was obtained by directly mixing BP nanosheets and CN (BP-to-CN ratios of 1:10). Afterwards, the mixture was subjected to a

thermal treatment at 300 °C for 2 h under N_2 atmosphere, thus obtaining BP-CN-*c*. Moreover, BP-CN-*c*-5 and BP-CN-*c*-20 were prepared by a similar procedure as BP-CN-*c* except using BP-to-CN ratios of 1:5 and 1:20, respectively. BP-CN-*c*-200 and BP-CN-*c*-400 were prepared by a similar procedure as BP-CN-*c* except using different annealing temperatures of 200 °C and 400 °C, respectively.

Preparation of BPC: BPC was prepared by directly mixing BP nanosheets with CNTs.

Stability test of BP and BP-CN-c: The exfoliated BP nanoflakes were dispersed into DI water with a concentration of 0.05 mg mL^{-1} and exposed to the ambient air. After 3 hours and 14 days, the dispersion was filtered and the filtrate was collected for inductively coupled plasma-optical emission spectroscopy analysis (ICP-OES) to detect the degraded P species (*e.g.,* H_3PO_3, H_3PO_4, or other P_xO_y species) dissolved in water. The stability of BP-CN-*c* was assessed in a similar method except using a dispersion concentration of 0.5 mg mL^{-1}.

Materials Characterization: The morphologies of samples were investigated by scanning electron microscopy (SEM, Zeiss Gemini 500) and transmission electron microscopy (TEM, Carl Zeiss Libra 200 Cs MC STEM). X-ray photoelectron spectroscopy (XPS) was performed by a Scienta Omicron Multiprobe system using a monochromatic Al Kα X-ray source and an Argus CU electron analyzer with a spectral resolution of 0.6 eV). X-ray diffraction (XRD) was measured by a PANalytical X'Pert Pro diffractometer with Cu-Kα radiation. Inductively coupled plasma-optical emission spectroscopy analysis (ICP-OES, Optima 2000 DV, *PerkinElmer Inc.*) was used to check the stability of BP and BP-CN-*c*. Barrett-Emmett-Teller (BET) surface area was measured from nitrogen adsorption/desorption isotherms at 77 K on a Quantachrome volumetric analyzer (Quadrasorb EVO/SI). Thermogravimetric analysis (TGA) was conducted using NETZSCH STA 409PC/PG. X-ray absorption near edge structure (XANES) spectra were performed at the Photoemission Endstation (BL10B beamline) of the National Synchrotron Radiation Laboratory (NSRL) in Hefei, China. FTIR measurements were carried out by a Bruker Optics ALPHA-E spectrometer equipped with Attenuated Total Reflectance (ATR) sample holder.

Electrochemical Characterization: All the electrocatalysts inks were prepared by thoroughly mixing 10 mg of catalysts in 980 μL of ethanol and 20 μL of 5% Nafion solution, and then the homogeneous catalysts inks were drop casted on the glassy carbon rotating disk electrode (RDE, 5 mm in diameter) with a loading of 0.5 mg cm^{-2} for electrocatalytic tests.

All the catalytic activities were measured on a PINE electrochemical workstation using a standard three-electrode configuration. RDE, Pt electrode, and a Hg/HgO electrode were used as the working electrode, counter electrode and reference electrode, respectively. In detail, the ORR polarization was conducted in O_2-saturated 0.1 M KOH solution with a scan rate of 5 mV s^{-1} and the OER polarization curves were obtained in N_2-saturated 1 M KOH solution with a scan rate of 5 mV s^{-1}. The accelerated durability test (ADT) was performed with potential cycles from 0.6 to 1.0 V *vs.* RHE for ORR and 1.3 to 1.7 V *vs.* RHE for OER with a sweep rate of 100 mV s^{-1}, respectively. The Nernst equation (equation S1) was used as the conversion formula for converting potentials from *vs.* Hg/HgO to *vs.* reversible hydrogen electrode (RHE).

$$E_{RHE}=E_{Hg/HgO}+0.059pH + 0.098 \qquad (S1)$$

The rotating ring-disk electrode (RRDE) measurements were used to determine the hydrogen peroxide yield ($\%H_2O_2$) and electron transfer number (n) during the ORR process according to equation S2-S3, where I_d is the disk current, I_r is the ring current, and N = 37% is the collection efficiency of the RRDE.

$$H_2O_2(\%) = 200 \times \frac{\frac{I_r}{N}}{I_d+\frac{I_r}{N}} \qquad (S2)$$

$$n = 4 \times \frac{I_d}{I_d+\frac{I_r}{N}} \qquad (S3)$$

The electrochemically active surface area of CN, BPC, BP-CN-*p*, and BP-CN-*c* were assessed by collecting CV curves with a small potential window of 1.1~1.2 V *vs.* RHE in N_2-saturated 0.1 M KOH solution.

***In-situ* Raman Spectro-Electrochemistry:** *In-situ* Raman spectro-electrochemistry was performed in a home-made three-electrode cell, in which BP-CN-*c* on a graphite electrode, platinum wire, and a Dri-Ref Ag/AgCl (3 M KCl) were used as the working electrode, counter electrode, and reference electrode, respectively. 1 M KOH was used as the electrolyte. Potential-dependent Raman spectra were recorded at potentiostatic steps starting from 1.0 to 1.6 V *vs.* RHE with a spectrum recorded at each step (0.2 V). Prior to that, Raman spectra of BP-CN-*c* at open circuit potential (0.875 V *vs.* RHE) was also recorded.

Raman measurements were performed using a S&I Monovista CRS + confocal Raman spectrometer at 442-nm excitation (Kimmon Koha IK4171I-G He-Cd laser) and 1500 grating.

Laser was adjusted and focused through a 40× immersion objective (Zeiss W Plan-APOCHROMAT; N.A. 1.0; WD=2.5 mm) to give an incident laser power of ~0.3 mW. Spectra were recorded for 30 s and 3 to 5 times accumulation. An Ivium potentiostat (IVA 25310) was used to apply the potential

Assembly of Zinc-Air Batteries: Zinc-air batteries were measured in a home-built electrochemical device. The electrocatalysts were drop casted on the carbon cloth (with a geometric area of 1.5 cm^2 and a loading amount of 1.0 mg cm^{-2}) attaching to the gas diffusion layer and used as air cathodes. Zinc foil was used as the anode, while 6.0 M KOH with 0.2 M zinc acetate was used as the electrolyte. Ni foam was used as the current collector for both anode and cathode. Electrochemical measurements were carried out with a CHI660E electrochemical workstation and a LAND CT2001A multi-channel battery testing system.

Computational Details: All density functional theory (DFT) based calculations were carried out using projected augmented wave (PAW)[2] pseudopotentials as implemented in the Vienna Ab-initio Simulation Package[3]. The Kohn-Sham orbitals were expanded in the plane-wave basis with the cutoff of 500 eV. The Perdew-Burke-Ernzerhof (PBE)[4] generalized gradient approximation (GGA) together with the D3 Grimme's dispersion corrections method[5] were employed to describe the exchange-correlation and van der Waals interactions. The structures were optimized until the force components were less than 0.01 eV/Å. To decouple the system with its periodic images, 15 Å of vacuum space was adopted along the z-axis. Brillouin zone integration was performed with a Γ-centered *k*-point mesh. Calculations were performed for pure black phosphorous, oxygen doped black phosphorous (BP-O), and graphitic carbon nitride (g-C_3N_4). The heterostructures were created for BP/g-C_3N_4 as well as BP-O/g-C_3N_4. Three rotation angles between BP (BP-O) and g-C_3N_4 (0°, 45° and 90°) were considered. The stacking structures are shown in Figure S10 and S11. The minimum total energy for BP/g-C_3N_4 as well as BP-O/g-C_3N_4 was obtained in the heterostructure with rotation angle of 45° and 0°. The charge density differences are calculated as the difference between the charge density of BP/g-C_3N_4, g-C_3N_4, and BP structures and visualized and plotted by VESTA[6].

The adsorption energies of oxygen-related intermediates on BP/g-C_3N_4 catalysts were calculated according to equation (S4-S6), where E* is the total energy of BP/g-C_3N_4 systems. E_{OOH}*, E_{OH}*, and E_O* are the total energies of the BP/g-C_3N_4 system with the adsorption of OOH, OH, and O, respectively. E_{H2O} and E_{H2} are the total energies of free H_2O and H_2 molecules in the gas phase. The Gibbs free energy (△G) of the OER/ORR intermediates can

be obtained from the computational hydrogen electrode (CHE) model shown in equation (S7),[7] where ΔE is the adsorption energy of an adsorbed species at each step. ZPE and ΔS are the difference of zero-point energies and the entropy between the adsorbed state and the free-standing state, respectively. T was set to room temperature (298.15 K).

$$\Delta E_O = E_O - E - [E_{H_2O} - E_{H_2}] \quad (S4)$$

$$\Delta E_{OH} = E_{OH} - E - [E_{H_2O} - 1/2\, E_{H_2}] \quad (S5)$$

$$\Delta E_{OOH} = E_{OOH} - E - [2E_{H_2O} - 3/2\, E_{H_2}] \quad (S6)$$

$$\Delta G = \Delta E + \Delta ZPE - T\Delta S \quad (S7)$$

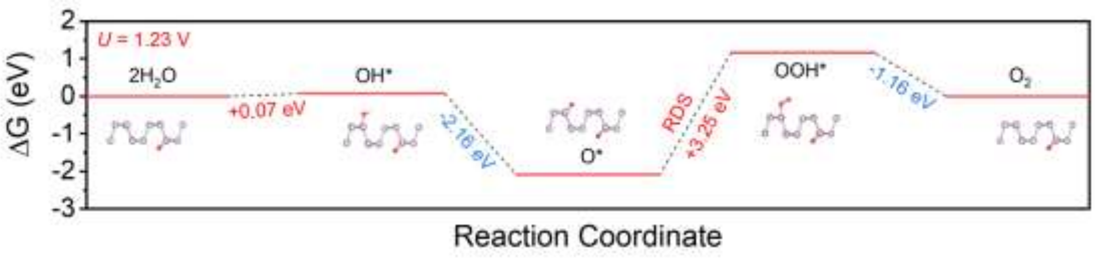

Figure S1. The calculated Gibbs free energy diagrams of the OER process on BP-O at U = 1.23 V.

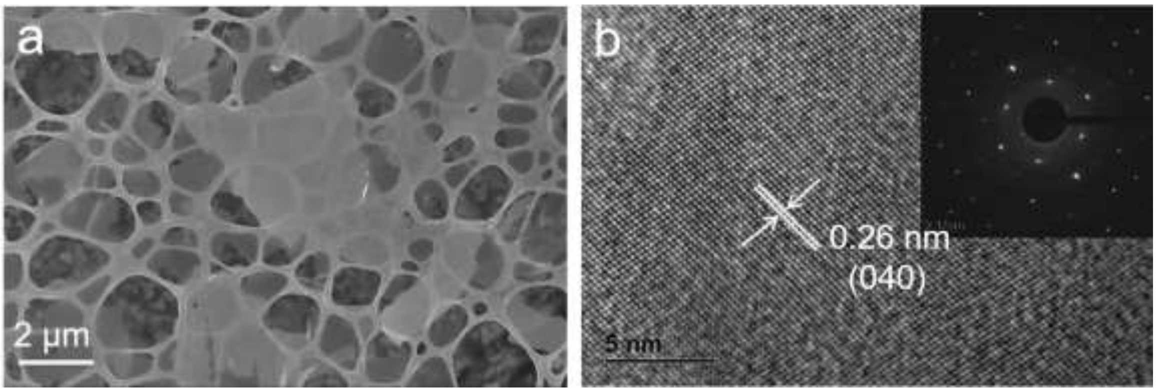

Figure S2. (a) SEM and (b) TEM images of 2D BP. Inset of (b) shows the selected area electron diffraction pattern.

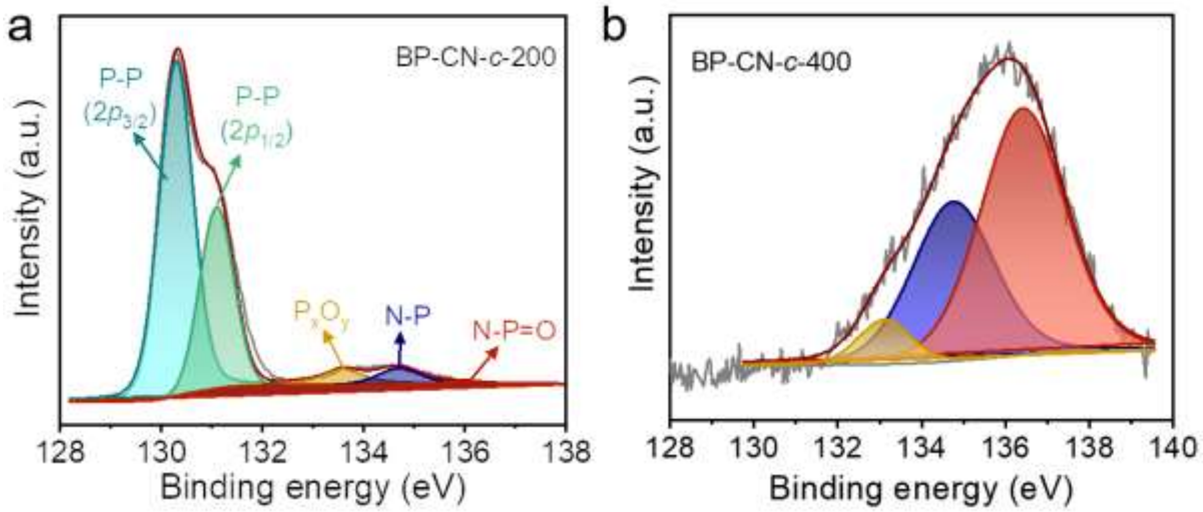

Figure S3. P 2p XPS of (a) BP-CN-*c*-200 and (b) BP-CN-*c*-400.

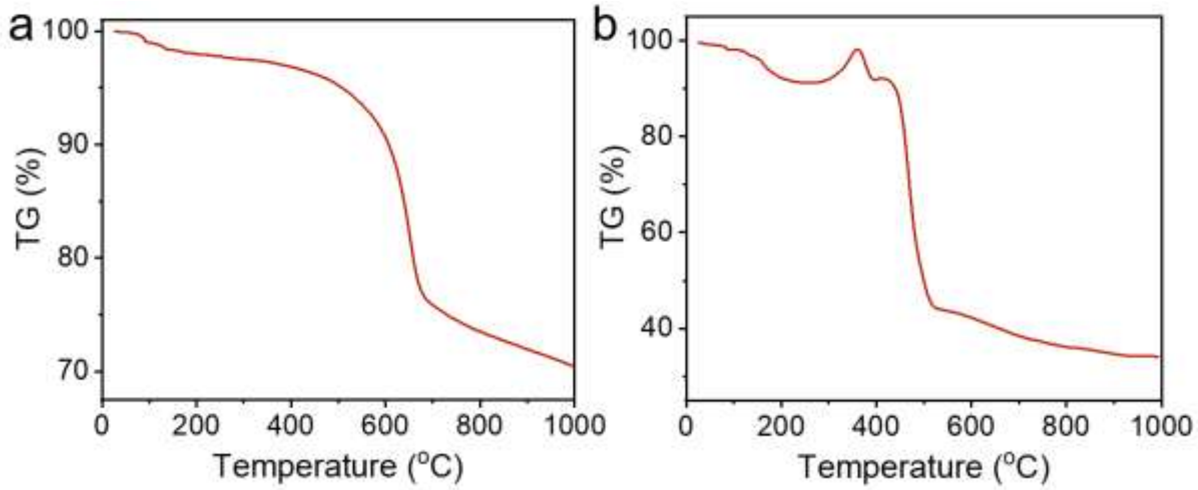

Figure S4. TGA curve of (a) CN and (b) BP in Ar atmosphere.

As revealed by Figure S4b, the decomposition of BP starts from about 400 ^{o}C, which indicates the annealing temperature should not be above 400 °C. P 2p XPS of BP-CN-*c*-200, BP-CN-*c*, and BP-CN-*c*-400 were used to check the formation of P-N covalent bonds in the three samples. In contrast with BP-CN-*c* (Figure 2a), BP-CN-*c*-200 have rare P-N bonds (Figure S3a), while BP-CN-*c*-400 (Figure S3b) shows no P-P peaks indicating the decomposition of BP. Therefore, we chose the annealing temperature of 300 °C to synthesize BP-CN-*c*.

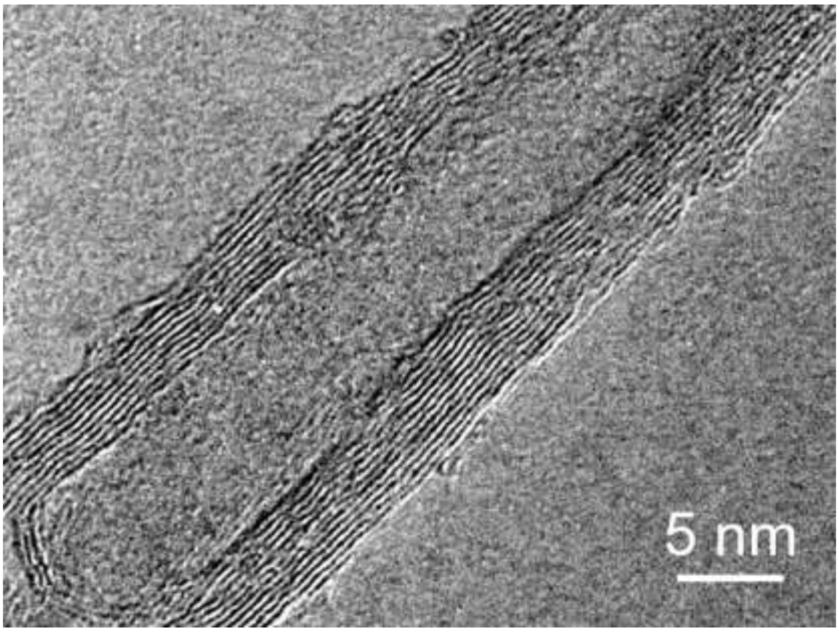

Figure S5. TEM image of CNTs.

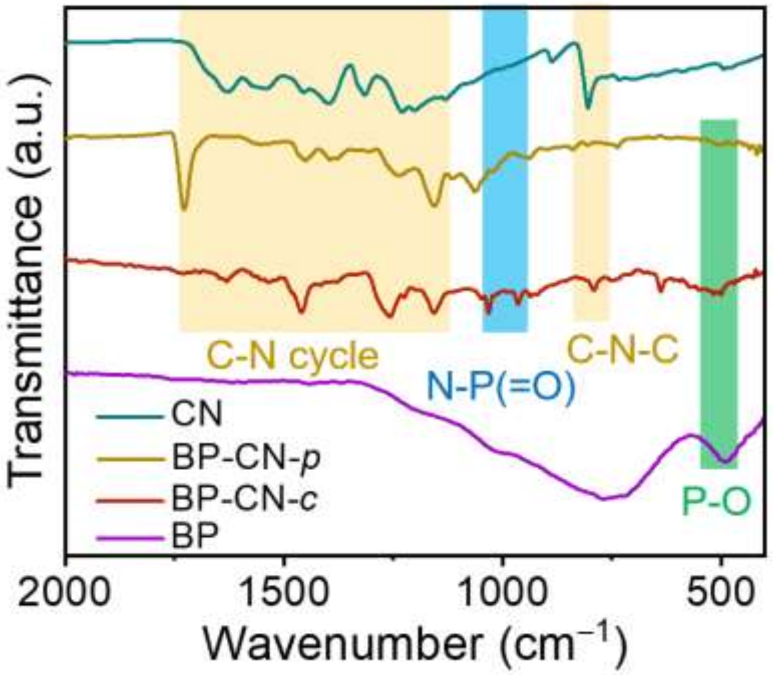

Figure S6. FTIR spectra of CN, BP-CN-*p*, BP-CN-*c*, and BP. Peaks ranging between 1200 and 1750 cm^{-1} are assigned to the typical stretching modes of C-N heterocycles. The peak located at 800 cm^{-1} is attributed to the out-of-plane bending vibration of characteristics of

triazine rings. The weak peak located at around 940-1070 cm^{-1} can be assigned to the P-N(=O) stretching mode, which is observed in the BP-CN-*c*.[8]

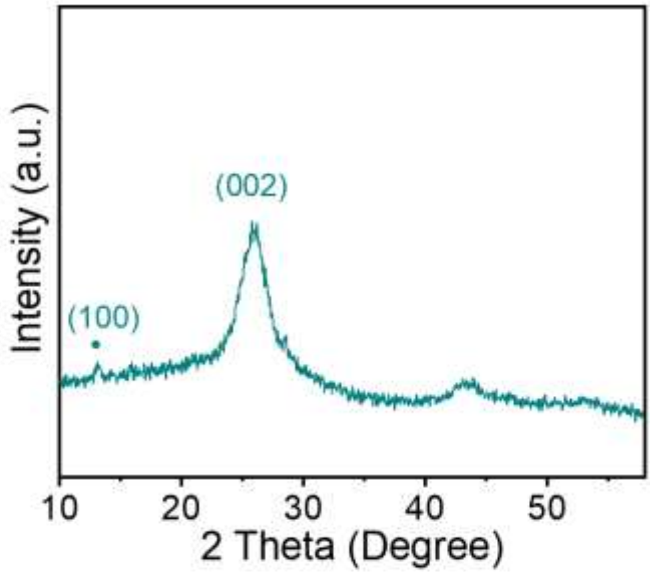

Figure S7. XRD spectra of CN. CN displays two obvious peaks at 2θ = 13.1° and 26.1°, which correspond to the (100) plane of g-C_3N_4 and the overlapped (002) planes of g-C_3N_4 and CNTs. The (100) plane of g-C_3N_4 refers to the repeated in-plane tri-s-triazine units.[9]

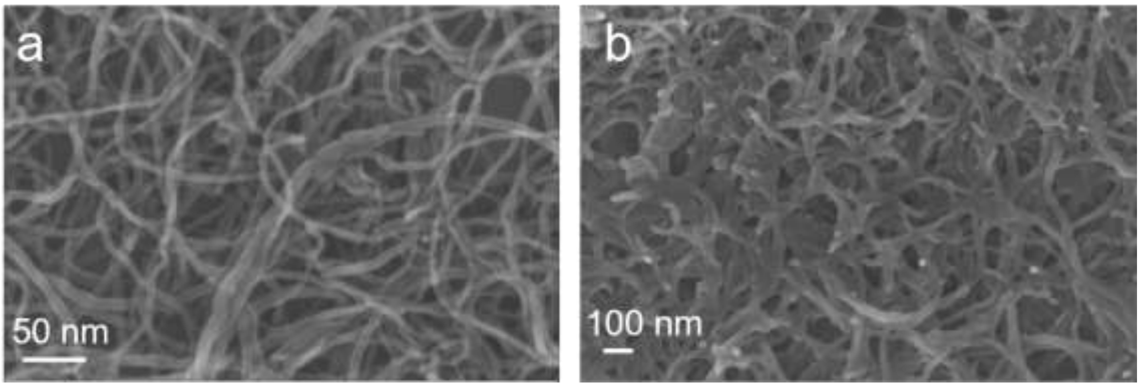

Figure S8. SEM images of (a) CNTs and (b) CN.

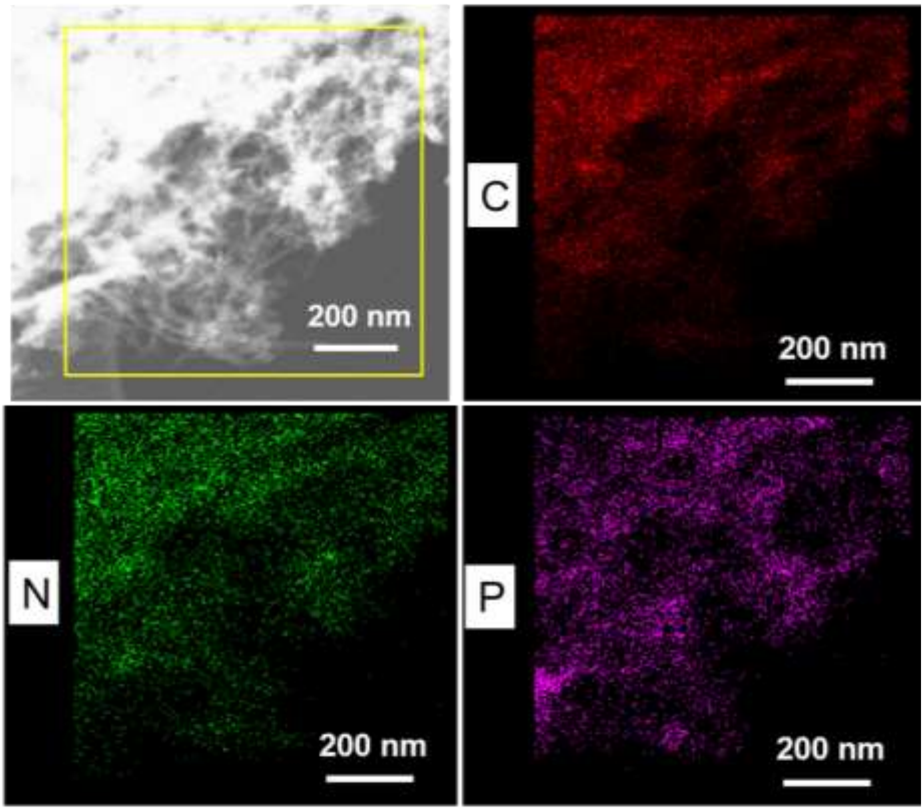

Figure S9. TEM image and the corresponding EDX spectroscopy elemental mappings of BP-CN-*c*.

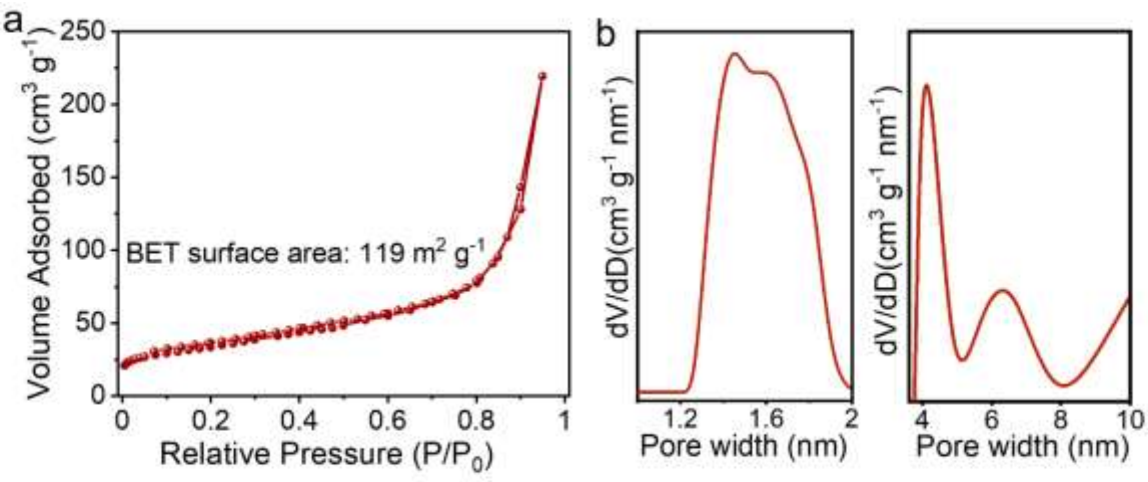

Figure S10. (a) N_2 adsorption-desorption isothermal curve and (b) pore size distribution of BP-CN-*c*.

The specific surface area of BP-CN-*c* was calculated to be 119 m^2 g^{-1}. Moreover, BP-CN-*c* exhibits micropores centered at around 1.5 nm and mesopores centered at 4.2 nm and 6.5 nm.

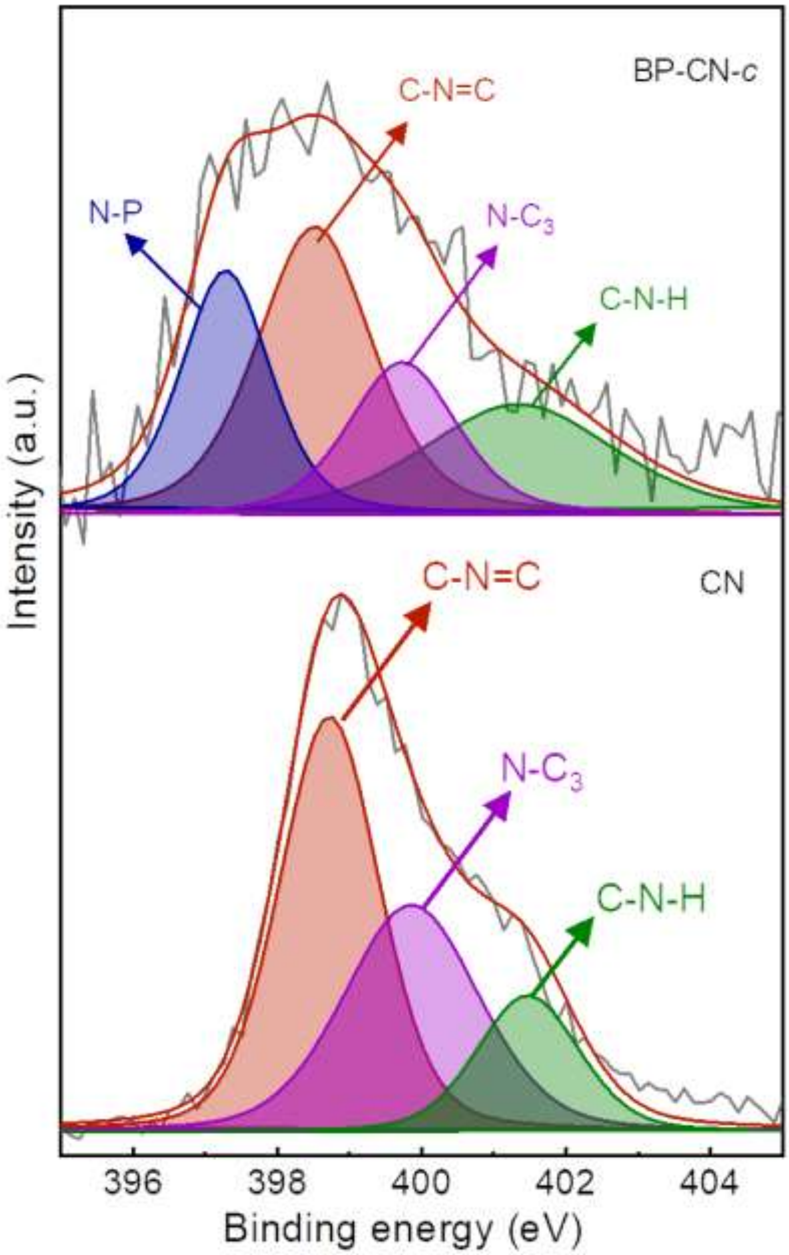

Figure S11. High-resolution N 1s XPS of CN and BP-CN-*c*. For the N 1s spectrum of CN, the three peaks at 398.7, 399.9, and 401.5 eV are attributed to the sp^2-bonded N (C−N=C), tertiary nitrogen N−C_3 groups, and amino groups (C−N−H) of g-C_3N_4, respectively. A new peak at 397.5 eV is detected for the N 1s spectrum of BP-CN-*c*, which can be assigned to the P-N bond.[10]

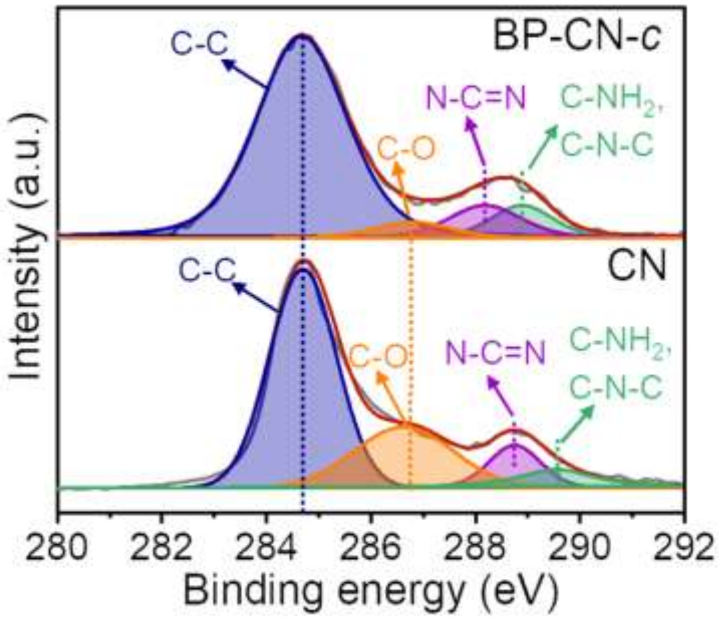

Figure S12. High-resolution C 1s XPS of CN and BP-CN-*c*.

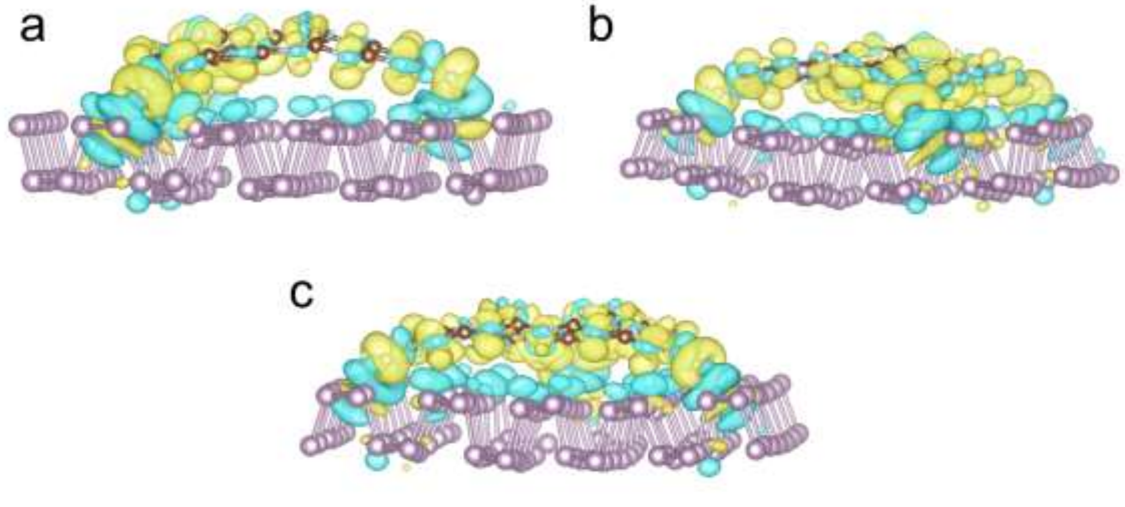

Figure S13. Electron density difference isosurfaces of the optimized BP/g-C_3N_4 hybrids with rotation of (a) 0 degree, (b) 45 degree, and (c) 90 degree between BP and g-C_3N_4. The purple, brown, and light blue balls represent P, C, and N atoms, respectively. Yellow and cyan regions correspond to electron accumulation and depletion, respectively (isosurface of 0.00185 e $Å^{-3}$).

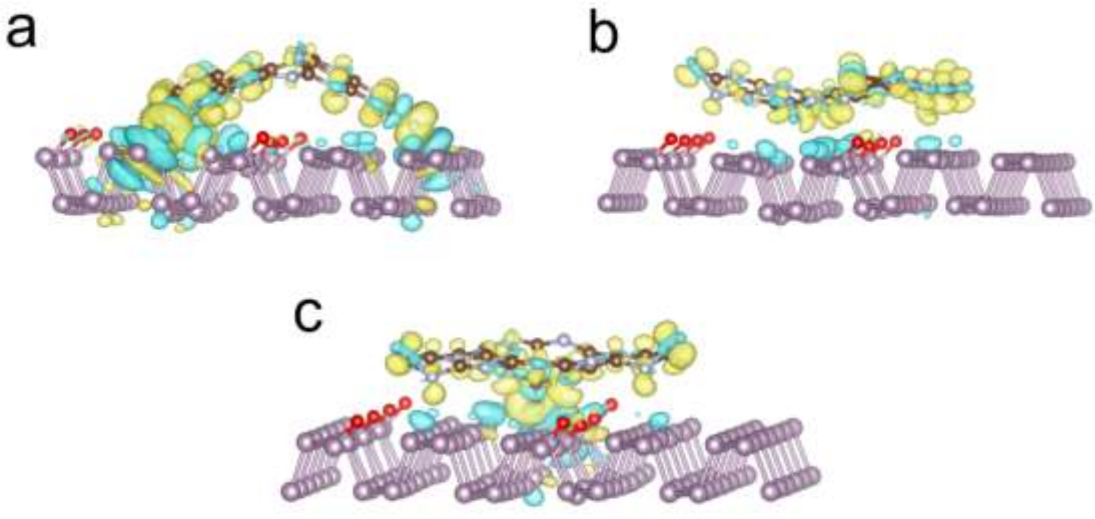

Figure S14. The electron density difference isosurfaces of optimized BP-O/g-C_3N_4 hybrids with rotation of (a) 0 degree (b) 45 degree and (c) 90 degree between BP-O and g-C_3N_4. The purple, brown, and light blue balls represent P, C, and N atoms, respectively. Yellow and cyan regions correspond to electron accumulation and depletion, respectively (isosurface of 0.00185 e $Å^{-3}$).

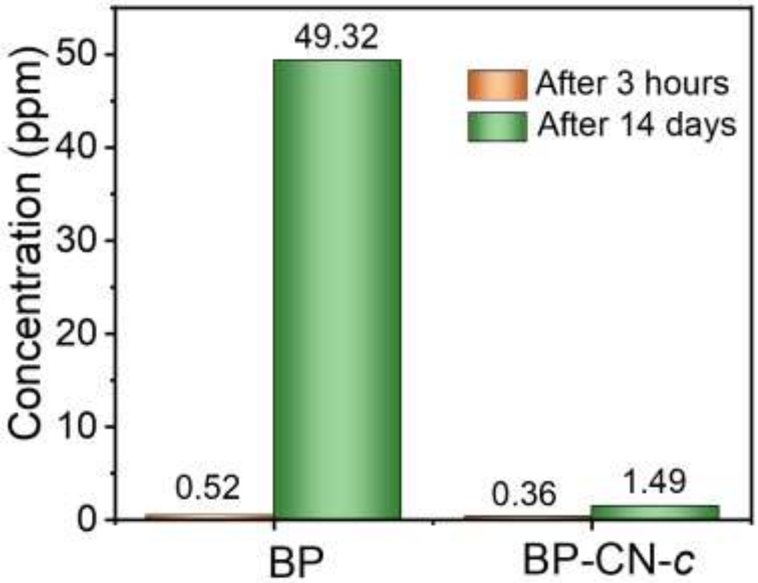

Figure S15. The concentrations of degraded P species dissolved in DI water analyzed by ICP-OES.

The stability of BP and BP-CN-*c* was evaluated by immersing them in DI water for two weeks. ICP-OES was employed to detect the degraded P species (*e.g.,* H_3PO_3, H_3PO_4, or P_xO_y species) dissolved in water. As shown in Figure S15, BP shows severe degradation with the highly increased P concentration (49.32 ppm), while BP-CN-*c* has slight degradation with the P concentration of 1.49 ppm.

Table S1. The elemental analysis of CN obtained with different urea-to-CNTs ratios.

CN samples	Weight percentage of N	Weight percentage of g-C_3N_4
CN8	2.5%	4.6%
CN16	8.4%	14.5%
CN24	15.0%	27.5%
CN32	18.7%	34.8%

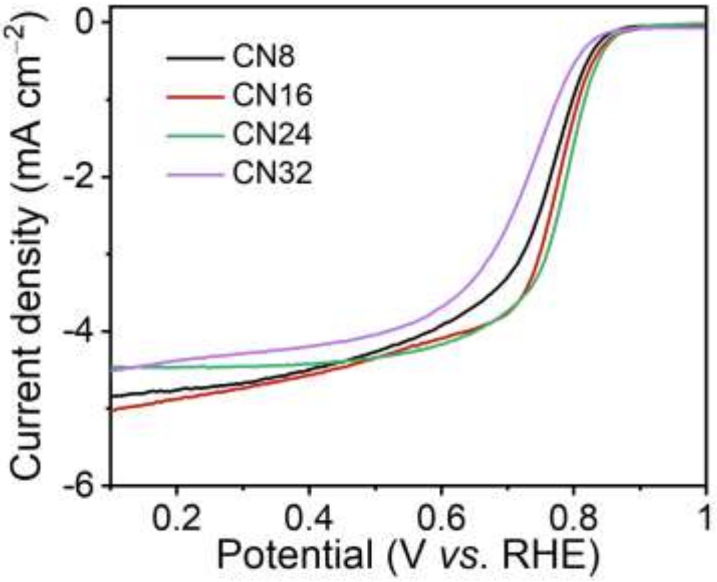

Figure S16. ORR polarization curves of CN8, CN16, CN24, and CN32.

Because g-C_3N_4 provides the main catalytic sites for ORR, the optimal CN sample was selected according to the ORR performance. As revealed, CN24 shows the highest ORR half-wave potential. Therefore, CN24 was further chosen to synthesize BP-CN-*c* and BP-CN-*p*.

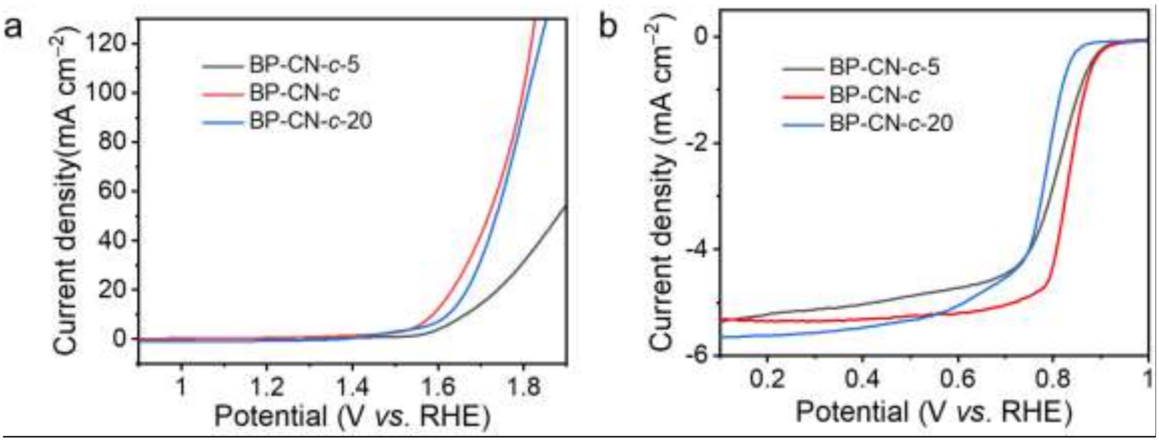

Figure S17. (a) OER and (b) ORR polarization curves of BP-CN-*c*-5, BP-CN-*c*, and BP-CN-*c*-20.

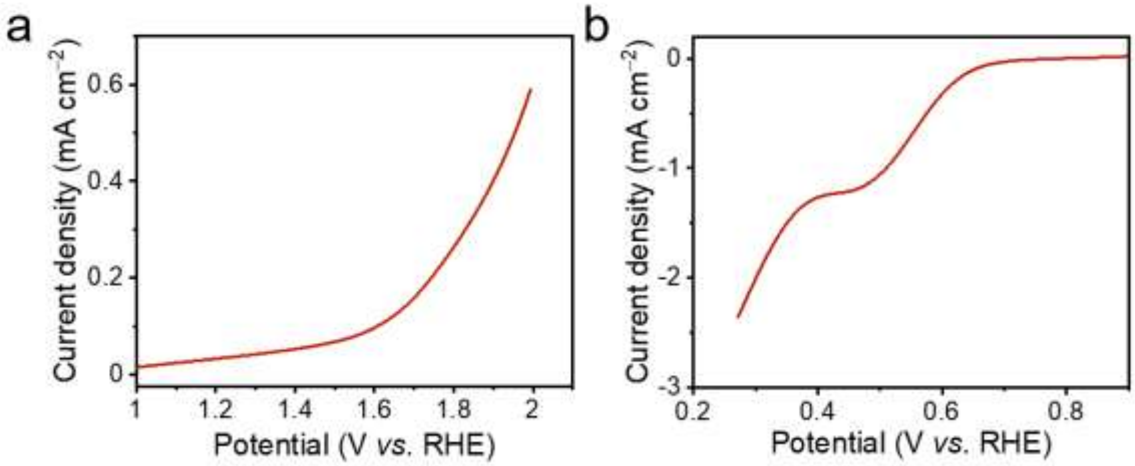

Figure S18. (a) OER and (b) ORR polarization curves of BP-g-C_3N_4.

Following the synthetic method of BP-CN-*c*, BP-g-C_3N_4 was prepared by replacing CN with g-C_3N_4. As revealed by the OER and ORR polarization curves, the sample shows very poor catalytic performance towards both OER and ORR. This can be assigned to the poor conductivity of BP-g-C_3N_4, which severely limits the electron transfer.[11]

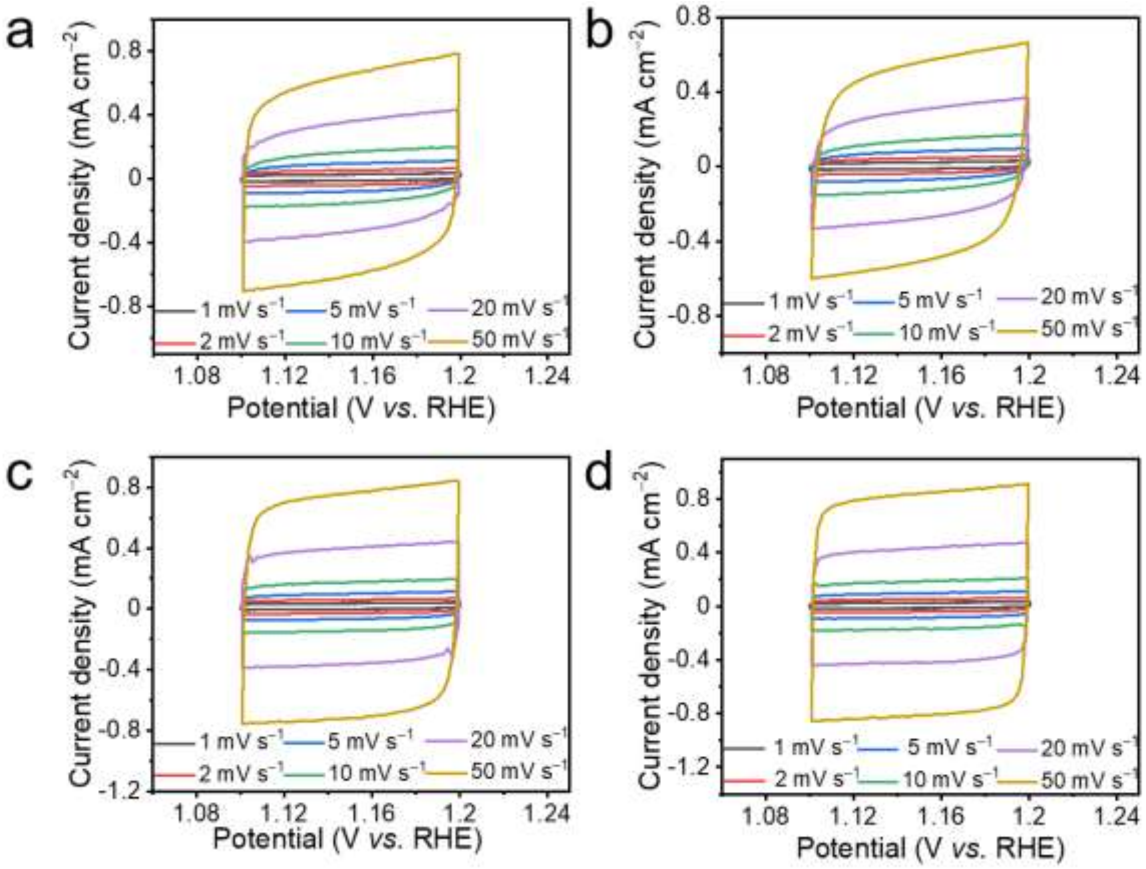

Figure S19. CV curves of (a) CN, (b) BPC, (c) BP-CN-*p*, and (d) BP-CN-*c*.

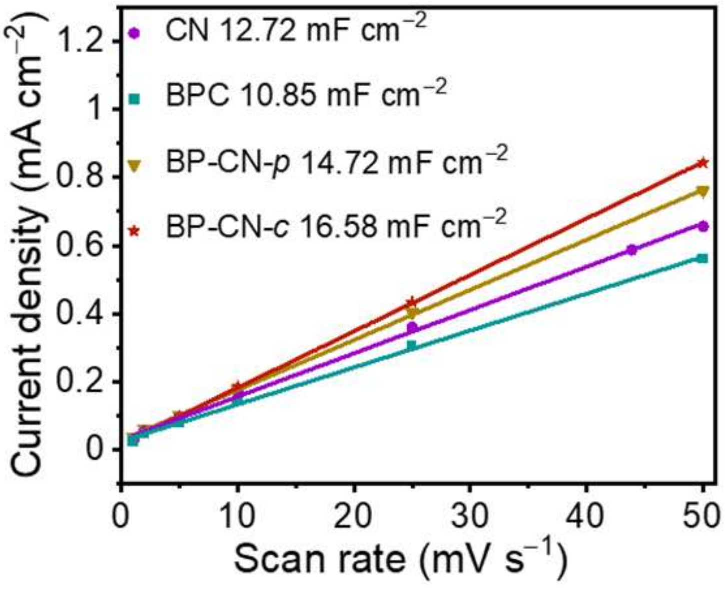

Figure S20. The electrochemical double-layer capacitances of CN, BPC, BP-CN-*p*, and BP-CN-*c*.

The electrochemically active surface area of CN, BPC, BP-CN-*p*, and BP-CN-*c* were assessed by collecting CV curves with a small potential window of 1.1~1.2 V *vs.* RHE. The CV curves of different samples are shown in Figure S19. As shown in Figure S20, BP-CN-*c* displays an electrochemical double-layer capacitance of 16.58 mF cm^{-2}, which substantially outperforms CN (12.72 mF cm^{-2}), BPC (10.85 mF cm^{-2}), and BP-CN-*p* (14.72 mF cm^{-2}). As the electrochemically active surface area is proportional to electrochemical double-layer capacitance, BP-CN-*c* has the highest electrochemical active surface area among all samples.

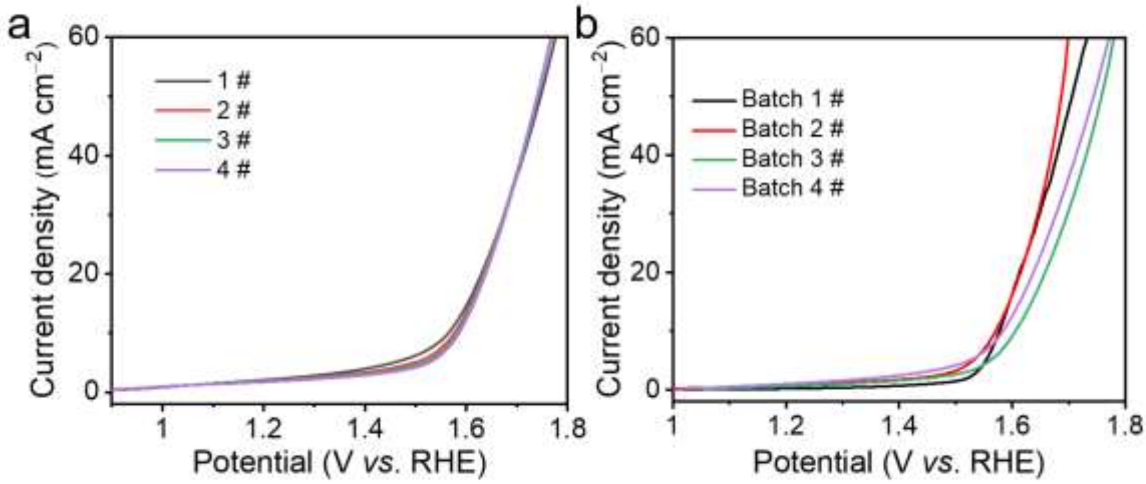

Figure S21. (a) OER polarization curves of four BP-CN-*c* electrodes prepared from the same batch. (b) OER polarization curves of BP-CN-*c* prepared from four batches.

To check the reproducibility of the BP-CN-*c* catalyst, four BP-CN-*c* based electrodes were prepared from the same batch. Polarization curves were collected to evaluate OER performance. Importantly, the BP-CN-*c* catalyst exhibit excellent reproducibility with similar OER overpotentials at 10 mA cm^{-2} (ranging from 333 to 354 mV). Moreover, four BP-CN-*c* samples prepared from different batches were also evaluated. Little variations among the four samples were observed for the OER overpotentials at 10 mA cm^{-2} (ranging from 340 mV to 370 mV).

Table S2. Comparison of the OER performance among BP-based electrocatalysts.

Catalyst	OER (overpotential at 10 mA cm^{-2})	Reference
BP-Ti	370 mV	[12]
BP nanosheets	≈ 570 mV	[13]
Co/BP	≈ 420 mV	[14]
Co_2P/BP	≈ 390 mV	[15]
S-doped BP nanosheets	410 mV	[16]
CoOx–BPQDs	360 mV	[17]
**BP-CN-*c***	**350 mV**	**This Work**

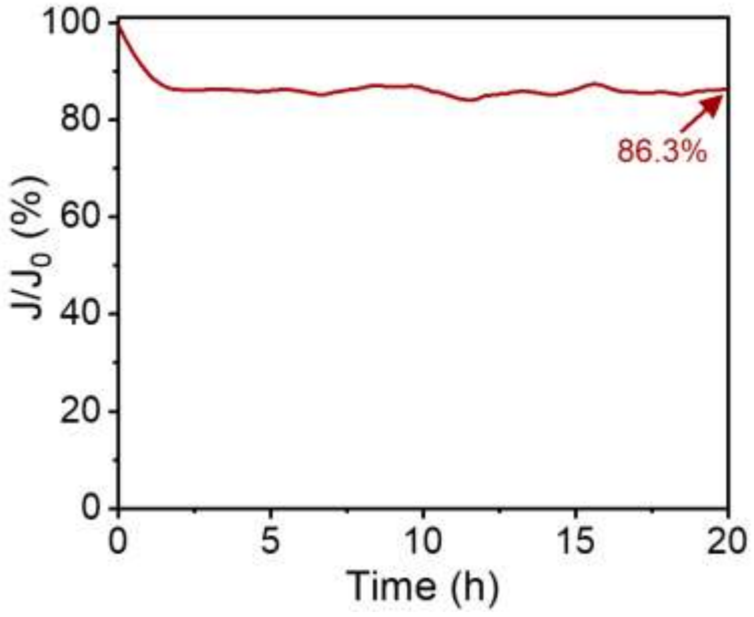

Figure S22. Chronoamperometric curve of BP-CN-*c* at 1.58 V *vs.* RHE for 20-h.

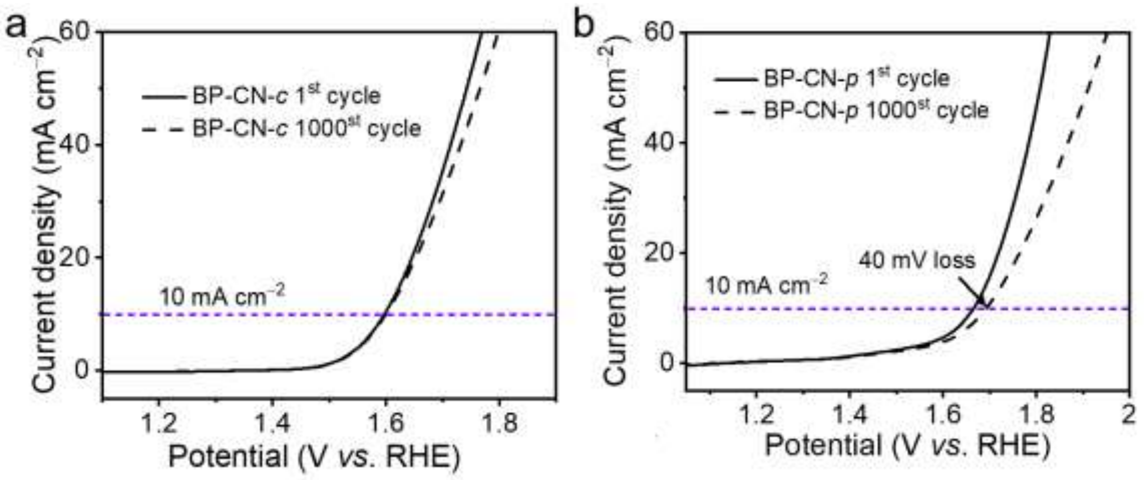

Figure S23. RDE polarization curves of (a) BP-CN-*c* and (b) BP-CN-*p* before and after 1000 cycles with a rotation speed of 1600 rpm and scan rate of 5 mV s^{-1}. ADT was carried out by cycling the potential between 1.3 and 1.7 V *vs.* RHE in a 1 M KOH solution for the evaluation of the long-term electrochemical durability.

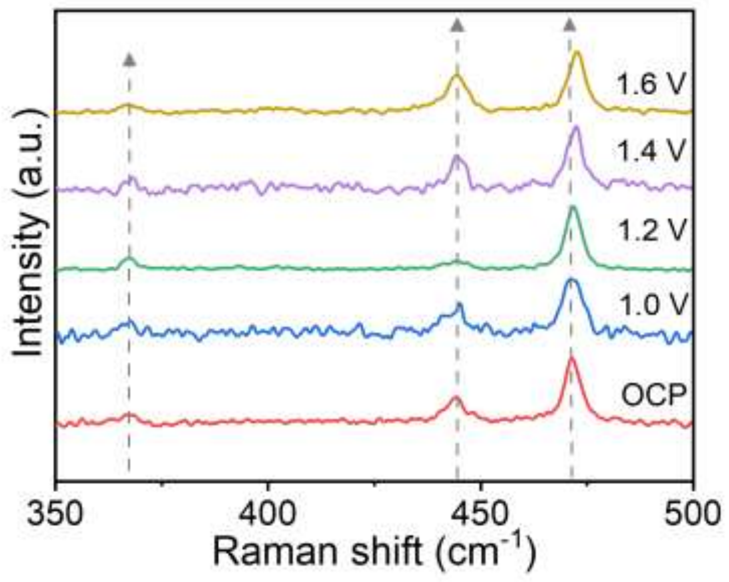

Figure S24. *In-situ* Raman spectro-electrochemistry for A_g^1, B_{2g} and A_g^2 vibrations of BP-CN-*c* at various potentials (*vs.* RHE). The peak intensity difference might be caused by the instability of the Raman laser and the bubble in the electrolyte.

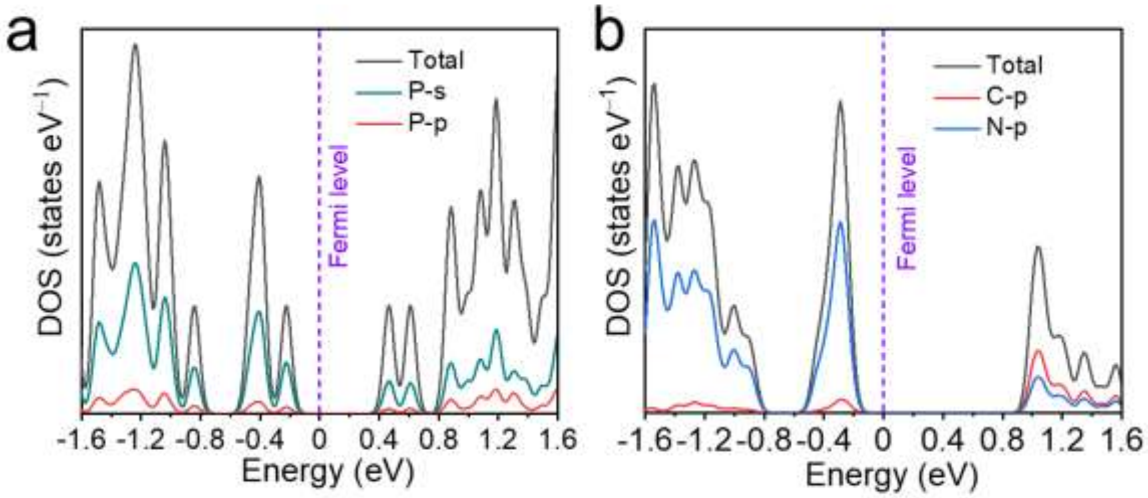

Figure S25. The calculated DOS of (a) BP and (b) g-C_3N_4.

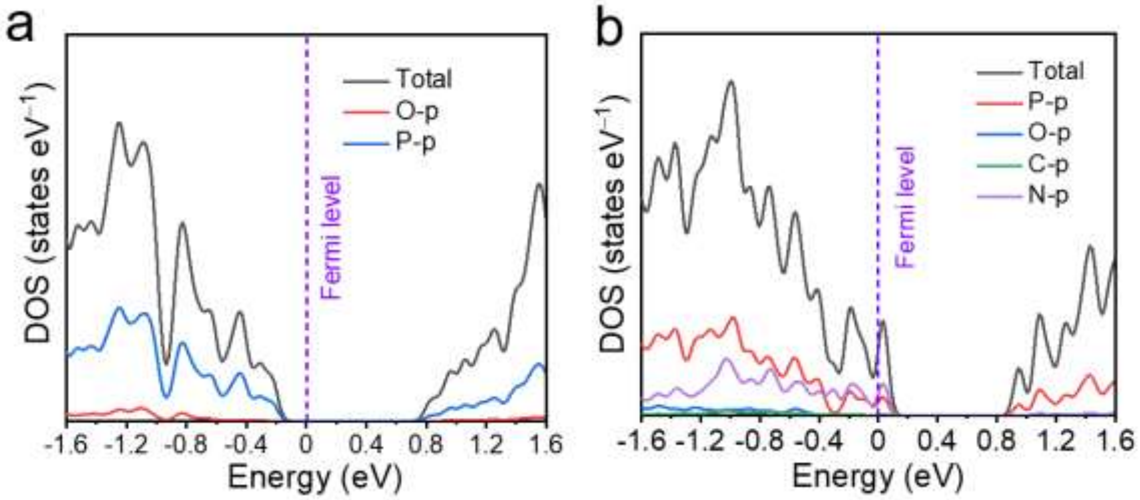

Figure S26. The calculated DOS of (a) BP-O and (b) BP-O/g-C_3N_4.

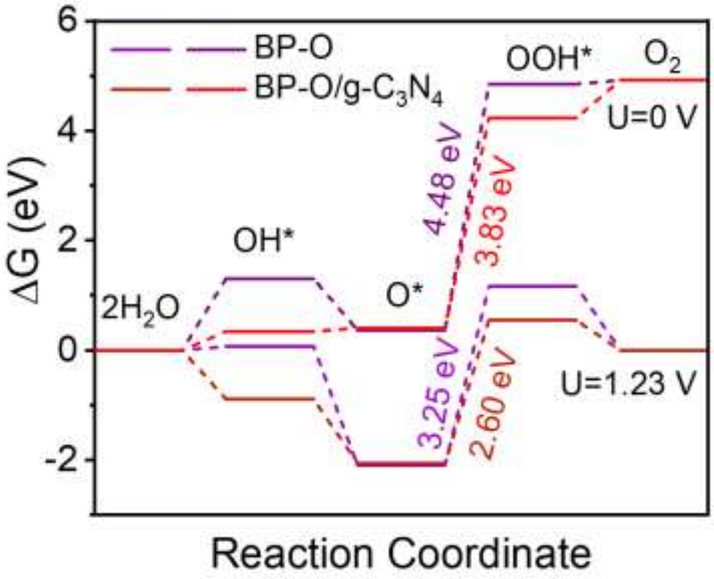

Figure S27. The free energy diagrams of the OER process on BP-O and the BP-O/g-C_3N_4 hybrid at U = 0 and 1.23 V.

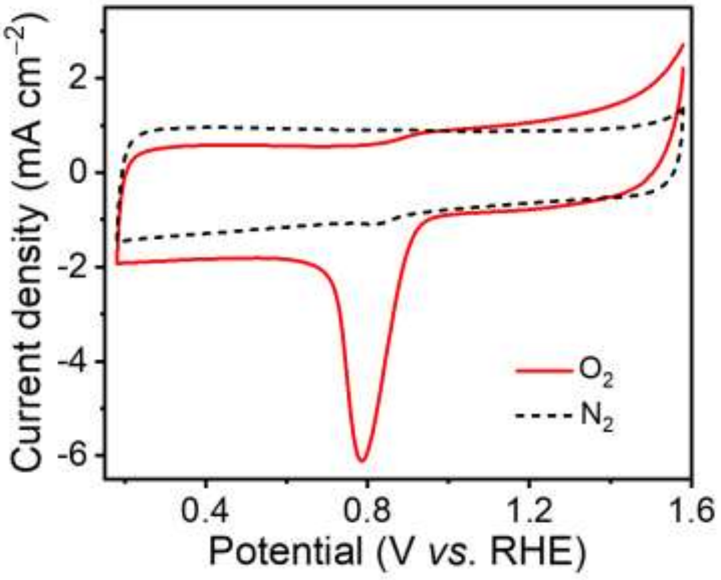

Figure S28. CV curves of BP-CN-*c* in N_2- and O_2-saturated 0.1 M KOH solutions.

CV curves of BP-CN-*c* were collected at a scan rate of 20 mV·s^{-1} in N_2- and O_2-saturated 0.1 M KOH solutions. The CV curve collected in the N_2-saturated electrolyte presents a quasi-rectangular shape without obvious redox peaks. In contrast, a distinct cathodic peak centered at ~0.80 V can be observed in the CV curve collected in the O_2-saturated electrolyte, indicating the pronounced electrocatalytic activity of BP-CN-*c* for ORR.

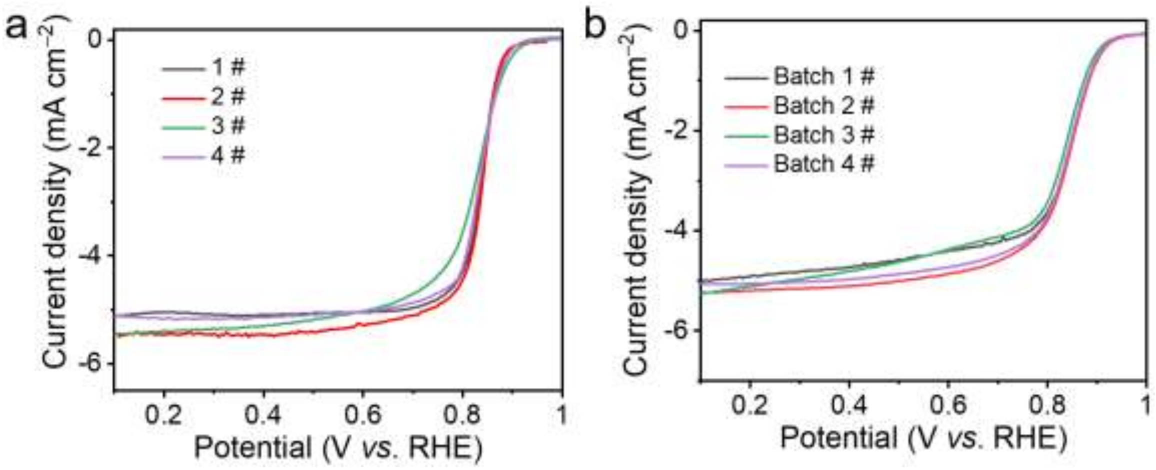

Figure S29. (a) ORR polarization curves of four BP-CN-*c* electrodes prepared from the same batch. (b) ORR polarization curves of BP-CN-*c* prepared from four batches.

To check the reproducibility of the BP-CN-*c* catalyst, four BP-CN-*c* based electrodes were prepared from the same batch. Polarization curves were collected to evaluate ORR performance. Importantly, the BP-CN-*c* catalyst exhibit excellent reproducibility with similar ORR half-wave potentials (ranging from 830 to 843 mV *vs.* RHE). Moreover, four BP-CN-*c* samples prepared from different batches were also evaluated. Little variations among the four samples were observed for the ORR half-wave potentials (ranging from 833 mV to 846 mV).

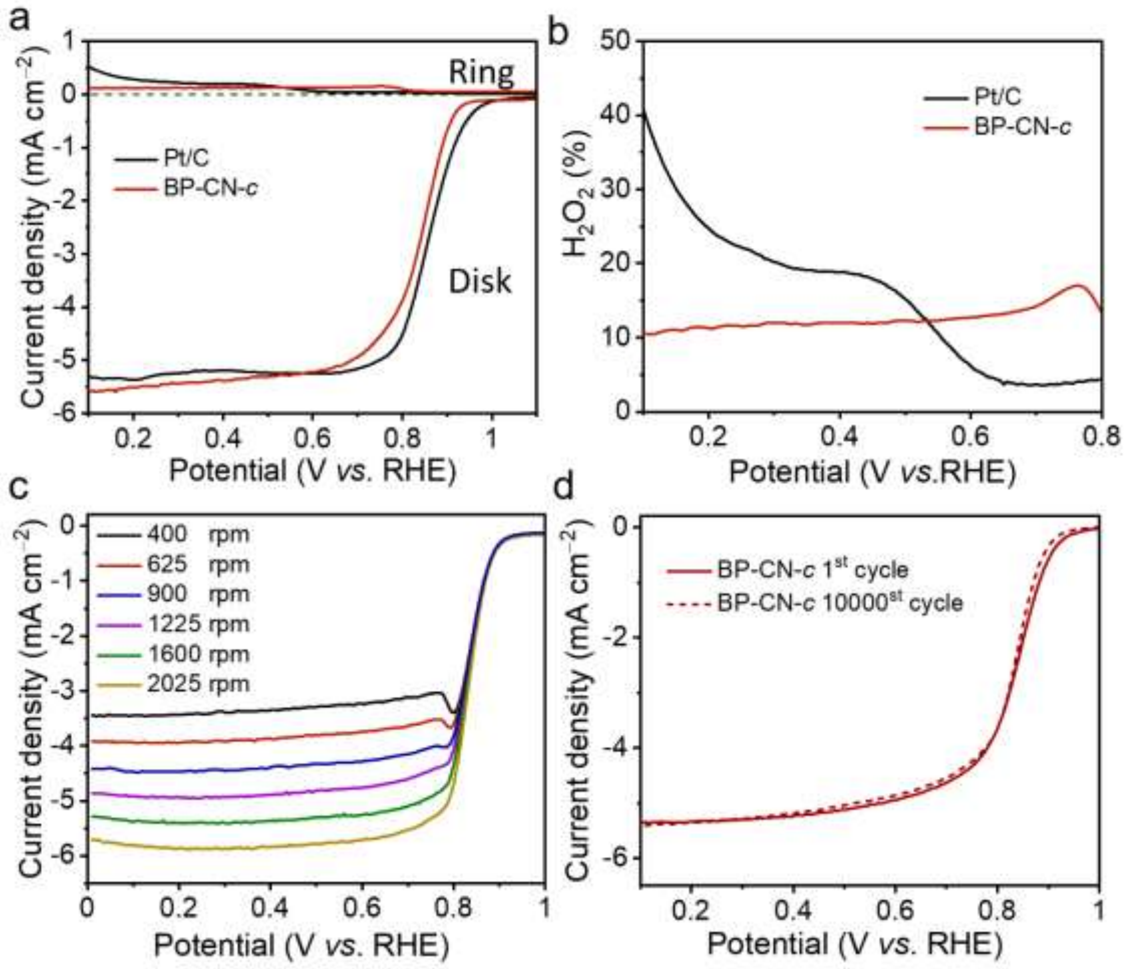

Figure S30. (a) RRDE curves, (b) H_2O_2 yields of the BP-CN-*c* and Pt/C catalysts calculated from the ring and disk currents. (c) RDE polarization curves of BP-CN-*c* in O_2-saturated 0.1 M KOH at rotation speeds ranging from 400 to 2025 rpm and a scan rate of 5 mV s^{-1}. (d) The RDE polarization curves of BP-CN-*c* before and after 10,000 cycles with a rotation speed of 1600 rpm at scan rate of 5 mV s^{-1}.

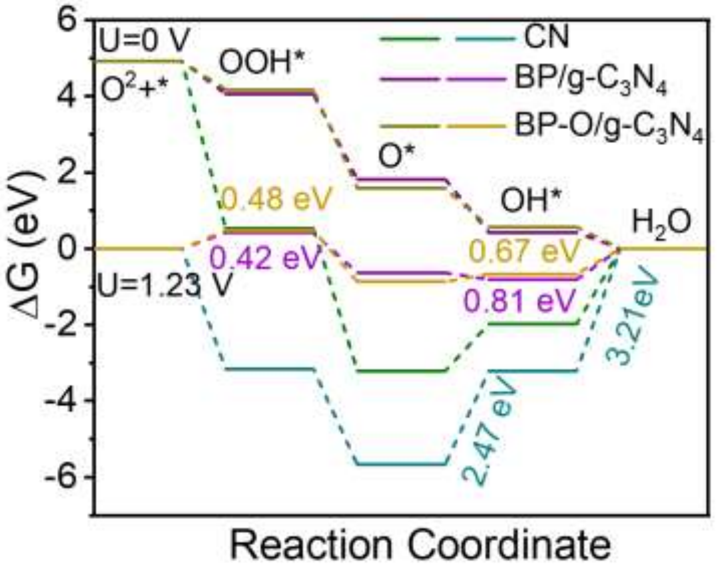

Figure S31. The free energy diagrams of the ORR process on CN, the BP/g-C_3N_4, and the BP-O/g-C_3N_4 at U = 0 and 1.23 V.

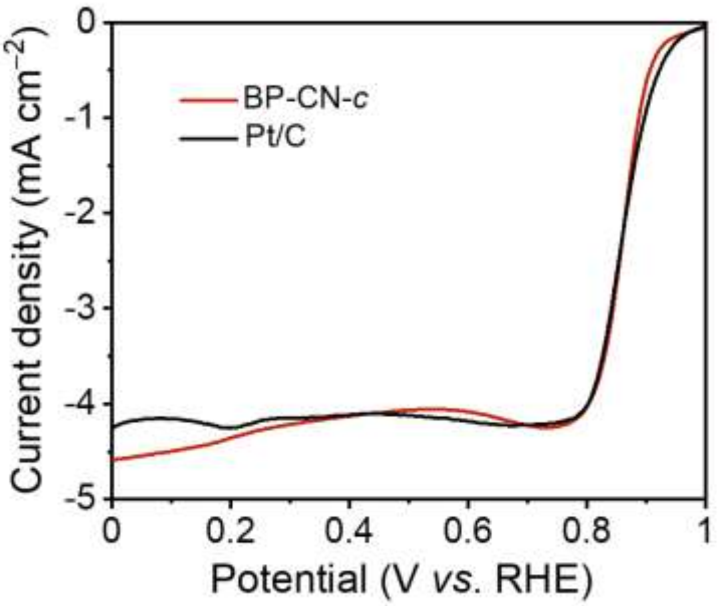

Figure S32. ORR polarization curves of BP-CN-*c* and Pt/C in 1 M KOH solution.

The half-wave potential of BP-CN-*c* is determined to be 0.86 V *vs.* RHE, which is close to the value calculated in 0.1 M KOH (0.84 V *vs.* RHE). This value was used to calculate ΔE, which is further compared with the state-of-the art metal-free catalyst (Figure 4d).

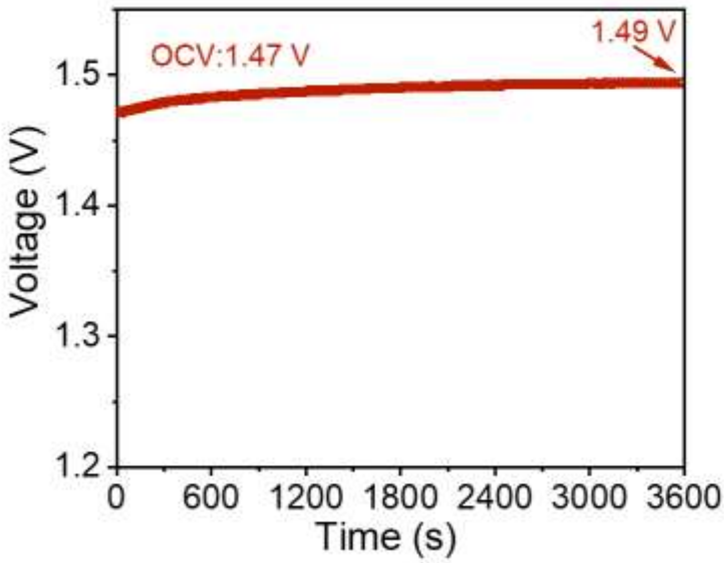

Figure S33. Open circuit voltage of BP-CN-*c* based ZABs.

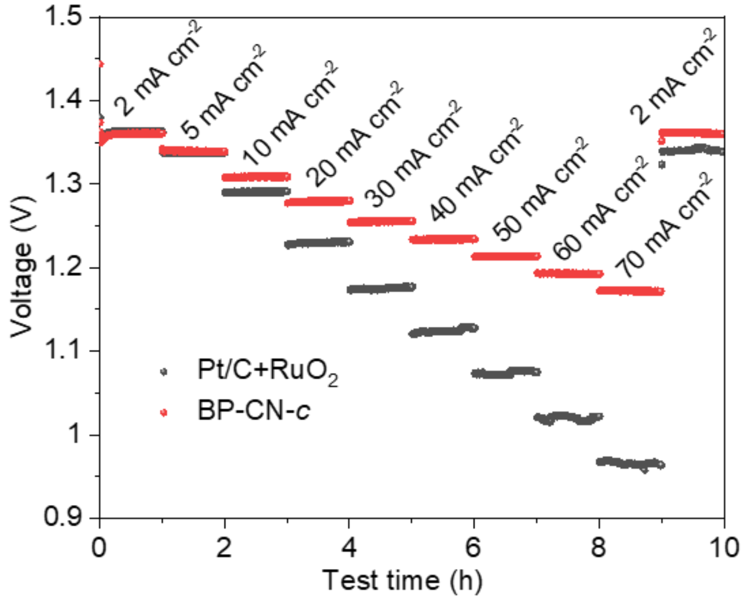

Figure S34. Discharging plateaus of BP-CN-*c* and Pt/C+RuO_2 based ZABs at different current densities.

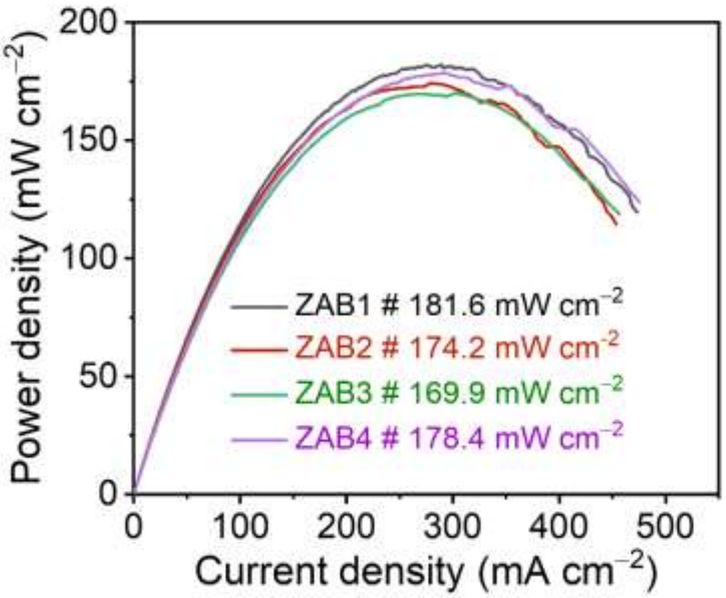

Figure S35. Power density curves obtained from four BP-CN-*c* based ZABs.

Four ZABs were fabricated with the BP-CN-*c* catalyst prepared from different batches, showing repeatable battery performance. For example, peak power densities of four ZABs are 181.6 mW cm^{-2}, 172.4 mW cm^{-2}, 169.9 mW cm^{-2}, and 178.4 mW cm^{-2}, respectively.

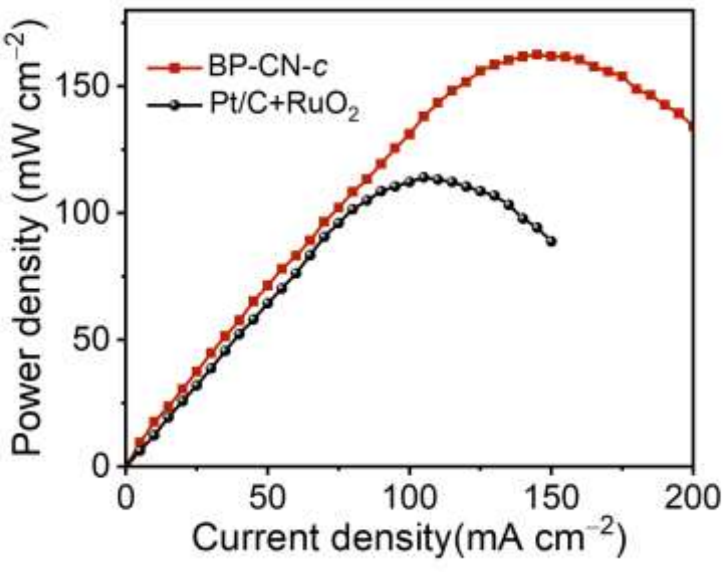

Figure S36. Power density curves of BP-CN-*c* and Pt/C+RuO$_2$ based ZABs derived from discharge/charge polarization curves.

The power density curves were derived from Figure 5b, which also indicate the superior performance of BP-CN-*c* based ZABs (162.4 mW cm^{-2}) compared with Pt/C+RuO$_2$ based ZABs (114 mW cm^{-2}).

Table S3. Performance comparison of various state-of-the-art ZABs.

**Bifunctional electrode**	**Power density**	**Open circuit voltage**	**Specific capacity**	**Energy density**	**Catalyst loading**	**Ref.**
Co_4N/carbon fibers/CC	174 mW cm^{-2}	1.4 V	774 mAh g^{-1} at 10 mA cm^{-2}	/	1 mg cm^{-2}	[18]
$NiCo_2S_4$/N-CNT	147 mW cm^{-2}	1.49 V	431.1 mAh g^{-1} at 10 mA cm^{-2}	554.6 Wh kg_{Zn}^{-1} at 10 mA cm^{-2}	1 mg cm^{-2}	[19]
Co/Co_3O_4@PGS	118.3 mW cm^{-2}	1.45 V	/	/	0.9 mg cm^{-2}	[20]
C-MOF-C_2 900	105 mW cm^{-2}	1.46 V	741 mAh g^{-1} at 10 mA cm^{-2}	/	0.5 mg cm^{-2}	[21]
$NPMC_{1000}$	55 mW cm^{-2}	/	735 mAh g^{-1} at 5 mA cm^{-2}	835 Wh kg_{Zn}^{-1} at 5 mA cm^{-2}	0.5 mg cm^{-2}	[22]
CuS/NiS_2	172.4 mW cm^{-2}	1.44 V	775 mAh g^{-1} at 5 mA cm^{-2}	1015.2 Wh kg_{Zn}^{-1} at 5 mA cm^{-2}	2 mg cm^{-2}	[23]
$Co_3FeS_{1.5}(OH)_6$	113.1 mW cm^{-2}	/	898 mAh g^{-1} at 20 mA cm^{-2}	/	0.5 mg cm^{-2}	[24]
NiO/CoN PINW	79.6 mW cm^{-2}	1.46 V	690 mAh g^{-1} at 5 mA cm^{-2}	945 Wh kg_{Zn}^{-1} at 5 mA cm^{-2}	/	[25]
$CuCo_2O_4$/N-CNT	83.8 mW cm^{-2}	1.36 V	817.4 mAh g^{-1} at 100 mA cm^{-2}	653.9 Wh kg_{Zn}^{-1} at 100 mA cm^{-2}	2.0 mg cm^{-2}	[26]
Ni-MnO/rGO aerogel	123 mW cm^{-2}	/	758 mAh g^{-1} at 5 mA cm^{-2}	930 Wh kg_{Zn}^{-1} at 5 mA cm^{-2}	10 mg cm^{-2}	[27]
$ZnCo_2O_4$/N-CNT	82.3 mW cm^{-2}	1.47 V	428.47 mAh g^{-1} at 10 mA cm^{-2}	595.57 Wh kg_{Zn}^{-1} at 10 mA cm^{-2}	2 mg cm^{-2}	[28]
Co-Nx-C	152 mW cm^{-2}	1.44 V	749.4 mAh g^{-1} at 20 mA cm^{-2}	840 Wh kg_{Zn}^{-1} at 20 mA cm^{-2}	0.5 mg cm^{-2}	[29]
CoPx@CNS	110 mW cm^{-2}	1.4 V	/	/	/	[30]
FeCo/Co_2P@NPCF	154 mW cm^{-2}	1.44 V	/	/	1 mg cm^{-2}	[31]
3DOM-Co@TiO_xN_y	110 mW cm^{-2}	1.466 V	697 mAh g^{-1}	865 Wh kg_{Zn}^{-1}	1.0 mg cm^{-2}	[32]
Co_3O_{4-x} HoNPs@HPNCS- 60	94.1 mW cm^{-2}	1.459 V	779.36 mAh g^{-1}	/	1 mg cm^{-2}	[33]
Co_2FeO_4 /NCNTs	90.68 mW cm^{-2}	1.43 V		605.1 Wh kg_{Zn}^{-1}	7.0 mg cm^{-2}	[34]
Fe_{20}@N/HCSs	140.8 mW cm^{-2}	1.57 V	726.9 mAh g^{-1}	/	1.0 mg cm^{-2}	[35]
**BP-CN-*c***	**168.3 mW cm^{-2}**	**1.47 V**	**793.9 mAh g^{-1} at 5 mA cm^{-2}**	**952.7 Wh kg_{Zn}^{-1} at 5 mA cm^{-2}**	**1.0 mg cm^{-2}**	**This Work**

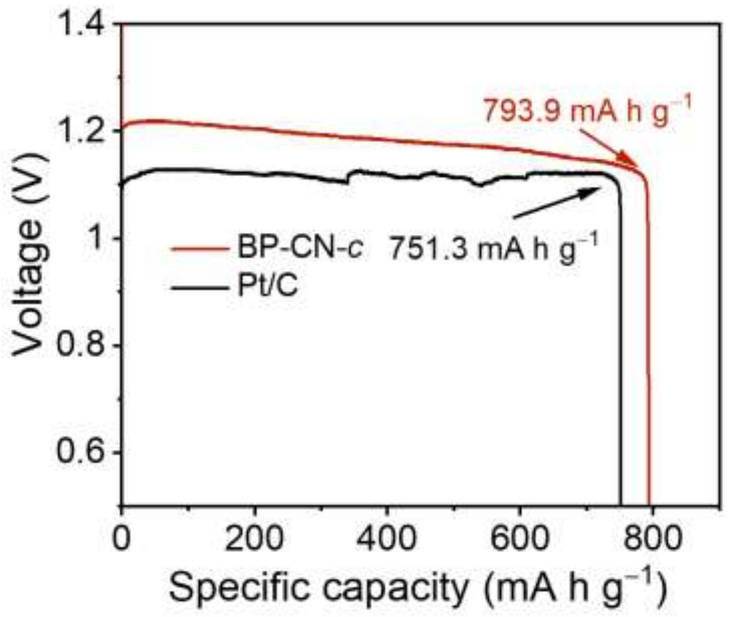

Figure S37. Discharge capacity plots of BP-CN-*c* and Pt/C based ZABs at 5 mA cm^{-2}.

References:

[1] S. Yang, K. Zhang, A. G. Ricciardulli, P. Zhang, Z. Liao, M. R. Lohe, E. Zschech, P. W. M. Blom, W. Pisula, K. Mullen, X. Feng, *Angew. Chem. Int. Ed.* **2018**, *57*, 4677.

[2] P. E. Blöchl, *Phys. Rev. B* **1994**, *50*, 17953.

[3] G. Kresse, J. Hafner, *Phys. Rev. B* **1993**, *47*, 558; G. Kresse, J. Furthmüller, *Comput. Mater. Sci.* **1996**, *6*, 15; G. Kresse, J. Furthmüller, *Phys. Rev. B* **1996**, *54*, 11169.

[4] J. P. Perdew, K. Burke, M. Ernzerhof, *Phys. Rev. Lett.* **1996**, *77*, 3865.

[5] S. Grimme, J. Antony, S. Ehrlich, H. Krieg, *J. Chem. Phys.* **2010**, *132*, 154104; J. Moellmann, S. Grimme, *J. Phys. Chem. C* **2014**, *118*, 7615.

[6] K. Momma, F. Izumi, *J. Appl. Crystallogr.* **2011**, *44*, 1272.

[7] J. K. Nørskov, J. Rossmeisl, A. Logadottir, L. Lindqvist, J. R. Kitchin, T. Bligaard, H. Jonsson, *J. Phys. Chem. B* **2004**, *108*, 17886.

[8] M. Zhu, S. Kim, L. Mao, M. Fujitsuka, J. Zhang, X. Wang, T. Majima, *J. Am. Chem. Soc.* **2017**, *139*, 13234; T. A. Luther, F. F. Stewart, J. L. Budzien, R. A. LaViolette, W. F. Bauer, M. K. Harrup, C. W. Allen, A. Elayan, *J. Phys. Chem. B* **2003**, *107*, 3168.

[9] D. Zhao, C. L. Dong, B. Wang, C. Chen, Y. C. Huang, Z. Diao, S. Li, L. Guo, S. Shen, *Adv. Mater.* **2019**, *31*, 1903545.

[10] T. Y. Ma, J. Ran, S. Dai, M. Jaroniec, S. Z. Qiao, *Angew. Chem. Int. Ed.* **2015**, *54*, 4646.

[11] J. Liang, Y. Zheng, J. Chen, J. Liu, D. Hulicova-Jurcakova, M. Jaroniec, S. Z. Qiao, *Angew. Chem. Int. Ed.* **2012**, *51*, 3892; Y. Zheng, Y. Jiao, J. Chen, J. Liu, J. Liang, A. Du, W. Zhang, Z. Zhu, S. C. Smith, M. Jaroniec, *J. Am. Chem. Soc.* **2011**, *133*, 20116.

[12] Q. Jiang, L. Xu, N. Chen, H. Zhang, L. Dai, S. Wang, *Angew. Chem. Int. Ed.* **2016**, *55*, 13849.

[13] X. Ren, J. Zhou, X. Qi, Y. Liu, Z. Huang, Z. Li, Y. Ge, S. C. Dhanabalan, J. S. Ponraj, S. Wang, *Adv. Energy Mater.* **2017**, *7*, 1700396.

[14] F. Shi, Z. Geng, K. Huang, Q. Liang, Y. Zhang, Y. Sun, J. Cao, S. Feng, *Adv. Sci.* **2018**, *5*, 1800575.

[15] W. Lei, G. Liu, J. Zhang, M. Liu, *Chem. Soc. Rev.* **2017**, *46*, 3492.
[16] Y. Chang, A. Nie, S. Yuan, B. Wang, C. Mu, J. Xiang, B. Yang, L. Li, F. Wen, Z. Liu, *Nanotechnology* **2018**, *30*, 035701.
[17] M. Batmunkh, M. Myekhlai, A. S. Bati, S. Sahlos, A. D. Slattery, T. M. Benedetti, V. R. Gonçales, C. T. Gibson, J. J. Gooding, R. D. Tilley, *J. Mater. Chem. A* **2019**, *7*, 12974.
[18] F. Meng, H. Zhong, D. Bao, J. Yan, X. Zhang, *J. Am. Chem. Soc.* **2016**, *138*, 10226.
[19] X. Han, X. Wu, C. Zhong, Y. Deng, N. Zhao, W. Hu, *Nano Energy* **2017**, *31*, 541.
[20] Y. Jiang, Y. P. Deng, J. Fu, D. U. Lee, R. Liang, Z. P. Cano, Y. Liu, Z. Bai, S. Hwang, L. Yang, *Adv. Energy Mater.* **2018**, *8*, 1870068.
[21] M. Zhang, Q. Dai, H. Zheng, M. Chen, L. Dai, *Adv. Mater.* **2018**, *30*, 1705431.
[22] J. Zhang, Z. Zhao, Z. Xia, L. Dai, *Nat. Nanotechnol.* **2015**, *10*, 444.
[23] L. An, Y. Li, M. Luo, J. Yin, Y. Q. Zhao, C. Xu, F. Cheng, Y. Yang, P. Xi, S. Guo, *Adv. Funct. Mater.* **2017**, *27*, 1703779.
[24] H. F. Wang, C. Tang, B. Wang, B. Q. Li, Q. Zhang, *Adv. Mater.* **2017**, *29*, 1702327.
[25] Z. Hu, Q. Li, B. Lei, Q. Zhou, D. Xiang, Z. Lyu, F. Hu, J. Wang, Y. Ren, R. Guo, *Angew. Chem. Int. Ed.* **2017**, *56*, 9131.
[26] H. Cheng, M. L. Li, C. Y. Su, N. Li, Z. Q. Liu, *Adv. Funct. Mater.* **2017**, *27*, 1701833.
[27] G. Fu, X. Yan, Y. Chen, L. Xu, D. Sun, J. M. Lee, Y. Tang, *Adv. Mater.* **2018**, *30*, 1704609.
[28] Z. Q. Liu, H. Cheng, N. Li, T. Y. Ma, Y. Z. Su, *Adv. Mater.* **2016**, *28*, 3777.
[29] C. Tang, B. Wang, H. F. Wang, Q. Zhang, *Adv. Mater.* **2017**, *29*, 1703185.
[30] C. C. Hou, L. Zou, Y. Wang, Q. Xu, *Angew. Chem. Int. Ed.* **2020**, *59*, 21360.
[31] Q. Shi, Q. Liu, Y. Ma, Z. Fang, Z. Liang, G. Shao, B. Tang, W. Yang, L. Qin, X. Fang, *Adv. Energy Mater.* **2020**, *10*, 1903854.
[32] G. Liu, J. Li, J. Fu, G. Jiang, G. Lui, D. Luo, Y. P. Deng, J. Zhang, Z. P. Cano, A. Yu, *Adv. Mater.* **2019**, *31*, 1806761.
[33] D. Ji, L. Fan, L. Tao, Y. Sun, M. Li, G. Yang, T. Q. Tran, S. Ramakrishna, S. Guo, *Angew. Chem. Int. Ed.* **2019**, *58*, 13840.
[34] P. Nakhanivej, X. Yu, S. K. Park, S. Kim, J.-Y. Hong, H. J. Kim, W. Lee, J. Y. Hwang, J. E. Yang, C. Wolverton, *Nat. Mater.* **2019**, *18*, 156.
[35] B. Wang, Y. Ye, L. Xu, Y. Quan, W. Wei, W. Zhu, H. Li, J. Xia, *Adv. Funct. Mater.* **2020**, *30*, 2005834.
